# From Dynamics to Diagnosis and Therapy: A Multiscale Computational Framework for MALT1-Targeted Cancer Theranostics

**DOI:** 10.1021/acs.jcim.6c01276

**Published:** 2026-07-30

**Authors:** Rodrigo M. Santos, Teodorico C. Ramalho

**Affiliations:** † Laboratory of Molecular Modelling, Department of Chemistry, 67739 Federal University of Lavras, Lavras 37200-000, Minas Gerais, Brazil; ‡ Centre for Basic and Applied Research, Faculty of Informatics and Management, University of Hradec Králové, Hradec Králové 500 03, Czech Republic

## Abstract

Cancer is one of the leading causes of death worldwide, making it a major concern in modern society. Therefore, the proposal of strategies against this illness is of major importance and has been widely studied by the scientific community in the past decades. In this sense, these strategies mainly focus on achieving improved therapy and diagnosis, with some proposals focusing on developing chemical agents capable of both treating and diagnosing cancer through targeting a cancer-related biomarker. In the role of cancer-related targets, MALT1 shows great potential as a cancer biomarker, being related to the nuclear factor-κB (NF-κB) signaling activation, an important biochemical process that regulates immune responses and inflammatory events in the human body, and its malfunction is related to the development and survival of several types of cancer. Despite MALT1’s importance as a cancer target, to the best of our knowledge, there is a lack of literature proposing chemical agents capable of treating and diagnosing cancer through this biomarker. Hence, the present work’s main goal was to propose a theranostic agent for this task, for which a combination of biased MD simulations, convolutional variational autoencoders (CVAEs), and quantum calculations was used. From this, it was possible to propose and optimize a compound to achieve a good MALT1 allosteric inhibition ratio while also presenting different fluorescence in different environments, crucial for signaling purposes. From this, both compounds **1** and **2** showed a cyan enol emission to blue enol emission when compared between the water-only environment and the protein environment, a feature that may serve as a molecular signature of the signaling process induced by the compound. In addition, this result also sheds light on the major relevance of enol emission of ESIPT-based probes, a few explored characteristics in the literature, which mainly focus solely on keto emission. Now, regarding inhibition, compound **1** indicated that the use of a phenothiazine derivative was a good choice, and by making a simple modification in its phenothiazine portion to generate compound **2**, the inhibition ratio more than doubled, from 29% for compound **1** to 71% for compound **2**, with compound **2** being an optimized chemical agent that has promising action suitable for theranostics purposes. Hence, the presented results show a promising direction toward the development of cancer theranostics drugs targeting the MALT1 allosteric pocket and provide a powerful theoretical framework that can be extended to other biological systems.

## Graphical abstract

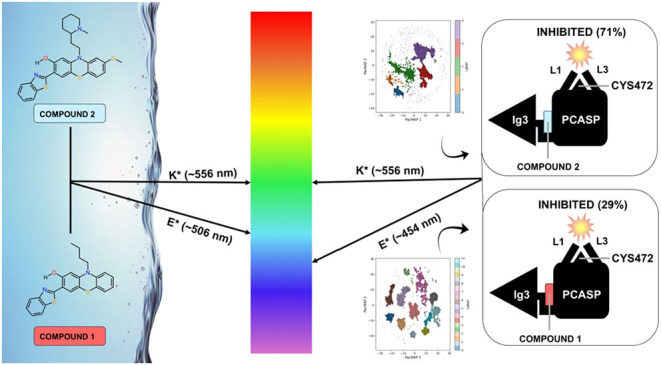

## Introduction

Cancer is a worldwide leading cause of death, causing nearly one in six deaths a year, with cancer being a generic term for a large group of diseases that share the characteristic of promoting uncontrollable cell growth and division.^1,2^ Given its critical importance in medicine, cancer diagnosis and therapy have been extensively investigated by the scientific community, leading to the development of numerous strategies over several decades of research.^3–5^ Hence, in the vast literature of strategies against cancer, one that draws the researchers’ attention is the usage of cancer-related proteins as targets for designed chemical compounds, such as the mucosa-associated lymphoid tissue lymphoma-translocation 1 (MALT1) protein.^6–10^

The MALT1 protein is a unique cysteine caspase-like protein, playing a major role in the nuclear factor-κB (NF-κB) signaling activation, a biochemical pathway that regulates immune responses and inflammatory events in various tissues of the human body.^11–14^ Due to this, MALT1 malfunction is associated with various types of cancer, mainly hematological cancers, but also extending to other types such as breast cancer.^13,15,16^ In addition, there is a relationship between MALT1 chronic activation and the survival of solid tumors, in which MALT1 protein showed a key function in the maintenance of an immunosuppressive tumor microenvironment.^8,17,18^

Due to its established role in cancer development and survival, MALT1 is considered a relevant molecular target for anticancer strategies. However, due to the complex structure of the MALT1 protein presenting disordered regions, especially in its caspase domain loops, its usage as a biological target is a laborious challenge for the scientific community.^19–22^ Despite that, previous works made an effort not only to advance in the comprehension of MALT1 inhibition in terms of its conformational diversity,^22,23^ but also to propose new inhibitory compounds for the MALT1 target, comprising not only a more traditional approach such as the development of substrate competitors,^7,24^ but also the development of compounds capable of allosterically inhibiting the MALT1 protein.^25–27^

Regarding the traditional approach of substrate competition, Rebeaud et al. (2008) developed the Z-VRPR-fmk irreversible peptide-based inhibitor, in which this compound mimics the substrate by binding to the catalytic cysteine located in the caspase domain of MALT1, generating an unavailable catalytic cysteine for substrate binding, inhibiting MALT1 functionality.^7,24^ Now, with respect to allosteric inhibition, MALT1 protein comprises an allosteric pocket located between its Ig3 and caspase domains, making it a different strategy of inhibiting MALT1, with the advantage of acting in a noncompetitive way, in which inhibition occurs by stabilizing MALT1 conformations with an unavailable catalytic cysteine.^23,25–27^ From this strategy, potent inhibitory compounds were proposed, such as the MLT-748 allosteric inhibitor,^28^ and the phenothiazine derivative compound, thioridazine, with other phenothiazine derivatives.^29^

In this context, the development of new therapeutic agents by targeting cancer-related biomarkers such as the MALT1 protein is of utmost value against cancer. However, modern strategies against cancer also focus on the development of an early and improved diagnosis, as therapeutic intervention in the early stage of the disease substantially increases the survival rate of patients affected by this illness.^30–32^ Thus, considering the importance of an improved diagnosis, the proposal of compounds in this direction can also be of much benefit.^33–35^ For this strategy, the aim is to find compounds capable of signaling cancer-related biomarkers, in which this signaling often takes advantage of spectroscopic phenomena, such as fluorescence.^33–36^ Hence, an important phenomenon for the design of such compounds is the excited-state intramolecular proton transfer (ESIPT) process, which consists in the proton transfer of a hydrogen bond donor group to a hydrogen bond acceptor group, a femtosecond scale process able to emit in two different wavelengths, one in ESIPT off (enol emission) and a different one for ESIPT on (keto emission), being it capable of signaling biological tissues through enzyme targeting.^37–40^

Considering, then, that both cancer therapy and diagnosis can be proposed by targeting cancer-related biomarkers, it is prudent to think that a much greater strategy against cancer is to unify both functionalities in one molecule, being a compound capable of treating and diagnosing at once by targeting a protein. Therefore, this is a popular approach against cancer in the literature, called theranostics, which consists in the development of chemical agents capable of both treating and diagnosing cancer through inhibition and signaling of a cancer-related protein, in which various types of compounds, inhibition, and signaling phenomena can be explored for such a goal.^41–44^ In this sense, the development of compounds capable of both inhibiting and signaling the MALT1 protein may be a promising strategy against cancer. However, despite theranostics being a popular approach against cancer, to the best of our knowledge, there is a lack of literature following this approach by using the MALT1 protein as a target.

Thus, the present work’s main goal consisted in the proposal of a compound that can act both as a therapy and a diagnostic agent against cancer by targeting the MALT1 protein, using for this an ESIPT-based phenothiazine-benzothiazole derivative molecule, an approach that has been shown to be a novelty in the literature. This proposal evokes strong theoretical technical challenges; however, previous works were able to build interesting frameworks and knowledge for evaluating ESIPT signaling capability and MALT1 inhibition.^22,23,40^ In this sense, by using sophisticated computational techniques such as neural networks and biased molecular dynamics (MD) simulations for biological systems conformational landscape exploration, combined with quantum methods for spectroscopic evaluation, such a proposal could be possible.^40,45–48^ This work introduces a powerful and transferable computational framework for the development of cancer theranostic agents, applicable to MALT1 and readily extendable to other protein targets, while also proposing a promising MALT1-targeted theranostic candidate.

## Materials and Methods

### Protein and Ligand Structures Preparation

As the protein’s initial structure for the theoretical investigations, the *Mus musculus* active MALT1 was used (PDB:3 V4L),^49^ once it presents fully crystallized catalytic site loops, while human MALT1 (PDB:3 V55)^49^ does not present a fully crystallized loop 3, making PDB:3 V4L more appropriate for computational allosteric inhibition investigations.^23,27^ In addition, the *Mus musculus* MALT1 presents a high homology with human MALT1 (PDB:3 V55),^49^ being 93%, and the same key catalytic site residues, being CYS472-ARG473-LYS474-ARG475 in the 3 V4L crystal, and CYS464-ARG465-LYS466-ARG467 in the 3 V55 crystal, are present.^23^ This makes it appropriate for a translational comprehension between the present work findings and those of human MALT1. Therefore, with the collected crystal, the covalent peptidic inhibitor Z-VRPR-fmk was removed, and the clean monomer was used.

Regarding the studied ligands, two compounds were proposed and compared as MALT1 allosteric inhibitors and probes, being then called compounds **1** and **2** throughout the entire manuscript for simplicity, and their structures are shown in Figure 1. Compound **1** is an already synthesized ESIPT-based probe,^50^ and compound **2** is a designed ligand based on the first compound, in which the benzothiazole portion was maintained, and its phenothiazine portion was changed to the thioridazine structure, an already reported structure with promising activity as a MALT1 allosteric inhibitor.^29^

**Figure 1:**
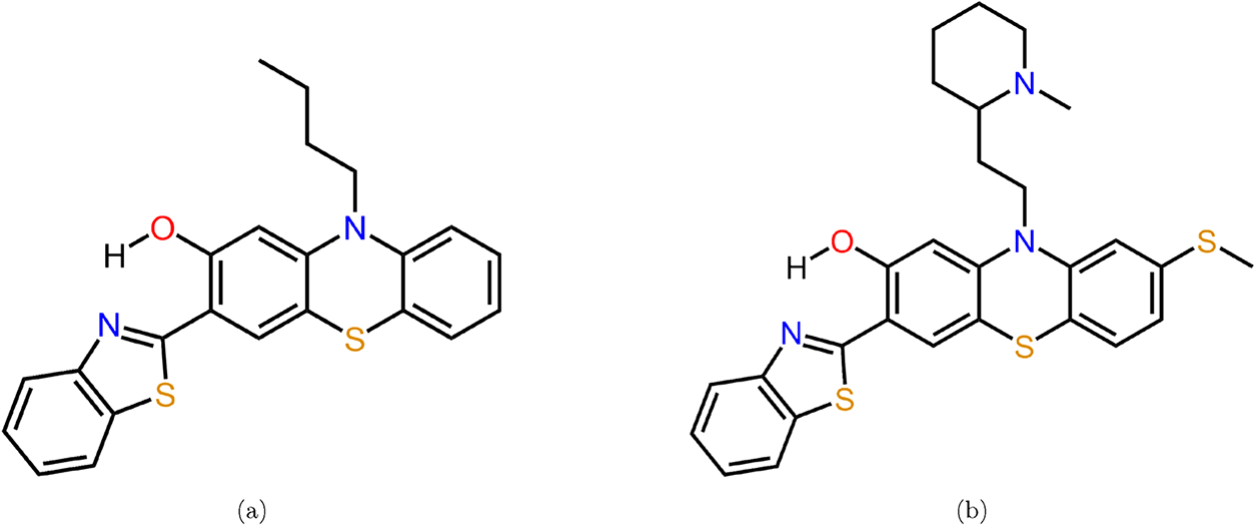
Chemical structure of (a) compound **1** and (b) compound **2**.

Both compounds **1** and **2** structures were prepared using GaussView 6 software,^51^ and optimized with Gaussian 09 software.^52^ For structure optimization, the B3LYP functional^53^ was used within the 6–311g basis set.^54^ Now, for generating the topology for the force field, the CGenFF web server^55^ was used.

### Generation of Protein–Ligand Complex Initial Structure

In order to obtain the protein–ligand complex structures with compounds **1** and **2** to use as starting structures for biased molecular dynamics (MD) simulations, docking calculations were performed to position the compounds in the allosteric pocket of the *Mus musculus* MALT1 clean monomer. For the calculations, nonpolar hydrogens were added to the protein structure to consider a physiological pH (∼7.4), and AutoDock Tools 1.5.7 ^56^ was used for this task. For the docking calculations, AutoDock Vina 1.1.2 software^57,58^ was used.

The calculations were performed considering a box (*x*,*y*,*z*) with dimensions of (20 Å, 20 Å, 20 Å), positioned in the allosteric pocket of MALT1. This calculation generated eight poses, in which the pose with lower energy was selected as the correct orientation of the respective compound in the MALT1 allosteric pocket. With this, the protein–ligand complex could be used as a starting structure for biased MD simulations.

### Biased Molecular Dynamics (MD) Simulations Setup

In total, 12 biased MD simulations were performed to investigate both the ESIPT on/off and inhibitory ratio. In this sense, four systems were studied, in which, for each of them, three replicas were simulated for 500 ns, totaling 1.5 μs simulated per system. The systems consisted of compound **1** in water-only (1.1), compound **1** allosterically positioned in MALT1 in a water reservoir (1.2), compound **2** in water-only (2.1), and compound **2** allosterically positioned in MALT1 in a water environment (2.2). For simulations, GROMACS 2023.3 ^59^ patched with PLUMED 2.9.0 ^60^ was used. The simulations followed the steps of minimization, NVT equilibration (a step in which different replicas are generated by changing the velocity generation seeds), NPT equilibration, and biased production. In addition, the system’s potential was described by using the CHARMM36 force field^61^ within the SPC water model.

Before the minimization step, the initial complex structures were inserted in a simulation box with a 10Å buffer size and water molecules, in which 13 sodium atoms were added to neutralize the −13 charge. With this, it was possible to proceed with energy minimization, and for this step the steepest descent integrator was used with a 5 fs time step, following until maximum force converged to values lower than 1000 kJ mol^–1^ nm^–1^. Once minimized, the system was submitted to NVT equilibration for 100 ps, in which the leapfrog integrator was used with a time step of 2 fs, using a temperature of 300 K and different velocity generation seeds for each replica. After the NVT equilibration, the system was submitted to NPT equilibration of 100 ps, using the same integrator and time step. For the NPT equilibration, C-rescale was used for pressure coupling, and a velocity rescaling thermostat was used for temperature coupling.

Once the systems were minimized and equilibrated, the biased production was performed for each simulation. For this step, the leapfrog integrator with a 2 fs time step was used. In addition, Parrinello–Rahman with isotropic pressure coupling was used within a compressibility factor of 4 × 10^–5^, and for temperature coupling, a velocity rescaling thermostat was used. Moreover, for neighbor searching and van der Waals interactions, the Verlet cutoff scheme was used with a cutoff of 12 Å, and long electrostatic interactions used particle mesh Ewald (PME) with the same cutoff.

Now, for biasing the production, the method used was the on-the-fly probability-enhanced sampling (OPES) expanded.^47^ For OPES usage, a multithermal expansion collective variable was settled by biasing the potential energy *U* to target the multicanonical ensemble over a temperature range of 300–400 K. Biasing the potential energy can be a good strategy when working with complex systems, since it avoids the choice of poor CVs.^47^ The defined pace of the bias potential update was 500 simulation steps (1 ps). For further analysis, the first 100 ns of each simulated replica were discarded in order to ensure bias convergence. The profiles of the free energy difference associated with reweighting the ensemble from the reference temperature (300 K) to 400 K show great stabilization after 100 ns (see Figure S2), and quantitative analysis of Δ*F*_400_ using blocks of 50 ns shows stabilization of its mean and standard deviation values after 100 ns, implying a stationary regime after 100 ns of the biased simulations (see Tables S1 and S2).

### ESIPT On/Off Distribution and TDDFT Calculations

The ESIPT on/off distribution was evaluated by observing the dihedrals 1 and 2 (see Figures 3A and 4) of the investigated compounds. From these ESIPT-related dihedral evaluations, the compound geometry could be evaluated in terms of its capability of performing ESIPT, and from the population analysis, an ESIPT on/off ratio could be calculated, this promising strategy being shown in previous work.^40^ Therefore, from the obtained distribution for compounds **1** and **2** in both water-only and protein environments, 12 metastable state clusters were labeled, and representative geometries were collected, with the conformation closest to the centroid of its respective metastable state being selected as representative.

Once the representative geometries of the accessed metastable states were obtained, the chemical environment (CE) was also collected and then submitted to quantum calculations to obtain the spectroscopic data related to the investigated compounds. As the CE of the water-only environment, it was collected with the compound and the water molecules within 5 Å of −OH and −N= groups of the benzothiazole portion, and as the CE of the protein environment, the two nearest amino acid residues of the same groups were collected, which can be seen in Table S3. For the calculations, Gaussian 09 software^52^ was used. For the ESIPT geometries, their configurations were changed from enol to keto by using the GaussView 6 ^51^ molecular editor; once these configurations had an appropriate geometry for ESIPT performance, according to the Eigen-Weller model.^40,62–64^

The quantum calculations followed two steps: the first one was a ground-state optimization step, and then a TDDFT step to obtain the emission data. For the ground-state optimization, the ONIOM procedure was used, in which the B3LYP functional^53^ with 6–311g basis set^54^ was used for the compound optimization layer, and the universal force field (UFF)^65^ was used for the CE optimization layer for the collected structures of all systems. Once ground-state optimization was done, the optimized structures were then used for the TDDFT calculations. For this step, the CAM-B3LYP functional^66^ was used with the same basis set as the previous step. The TDDFT calculations were performed considering the first excited state (root = 1), giving the first three singlet states, in which the one with higher harmonic oscillator strength was considered as the most probable, and its emission wavelength was collected and reported.

### CVAE Architecture and Protein Conformational Landscape Generation

The use of embedding techniques has been shown to be of great value when studying protein conformational dynamics, especially when using artificial neural networks (ANN) with autoencoder architectures.^45,46,67–69^ Hence, its usage for MALT1 allosteric inhibition comprehension also showed to be a good strategy, being capable of identifying metastable states and generating a conformational landscape for investigation.^23^ In this sense, the present work used a sophisticated ANN architecture called Convolutional Variational Autoencoder (CVAE), which showed enormous potential in generating conformational landscapes of protein dynamics,^45,67^ making it of great value for accessing and evaluating allosteric inhibition in MALT1.

The use of 2D CVAEs requires the use of images/maps as input data; hence, MALT1 conformations should be described with this type of data in order to generate its conformational landscape by using a 2D CVAE. In this sense, MALT1 contact maps were constructed from the obtained trajectories, in which their generation consisted of first calculating a pairwise distance matrix of Cα atoms and second filtering the distance matrix with a chosen cutoff in order to generate a binary contact map (a matrix with 1 and 0 only). Therefore, for calculating the distance matrix, Cα atoms of residues 360–371 (Loop 3), 501–517 (Loop 1), and 471–473 (key MALT1 catalytic residues) are used, generating a 32 × 32 matrix. In previous work, Santos et al.^23^ demonstrated the great importance of loops 1 and 3 in MALT1 allosteric inhibition; thus, this work focused its allosteric inhibition investigation on these key catalytic site chains, in which the usage of Cα atom contact maps can appropriately provide a deep clustered conformational landscape for further inhibition evaluation, with a feasible computational cost.^45^ Now, for creating the contact maps, a cutoff of 8 Å was used, in which from a search in the distance matrix, for every distance below 8 Å, 1 is assigned (there is contact between atoms *i* and *j*), and for every value above 8 Å, 0 is assigned (there is no contact between atoms *i* and *j*), generating the binary contact map that will feed the used 2D CVAE. In total, for each system consisting of 3 replicas, 60003 contact maps were constructed to describe the explored conformational dynamics through biased MD simulations.

Regarding the used 2D CVAE architecture, Figure 2 shows its scheme with the used 2D convolutional layers, the latent space generation, and contact map reconstruction. In addition, Table 1 shows the hyperparameters used for the 2D CVAE architecture. For the 2D CVAE training, a train-test using 20% of samples for internal validation and 80% for training was used. Moreover, 10 samples were separated for further external validation in order to evaluate the contact map reconstruction. Regarding the training, a learning rate of 0.001 was used with a batch size of 32 and trained for 100 epochs. For the construction and training of the used 2D CVAE, PyTorch 2.2.2 ^70^ library was used.

**Figure 2:**
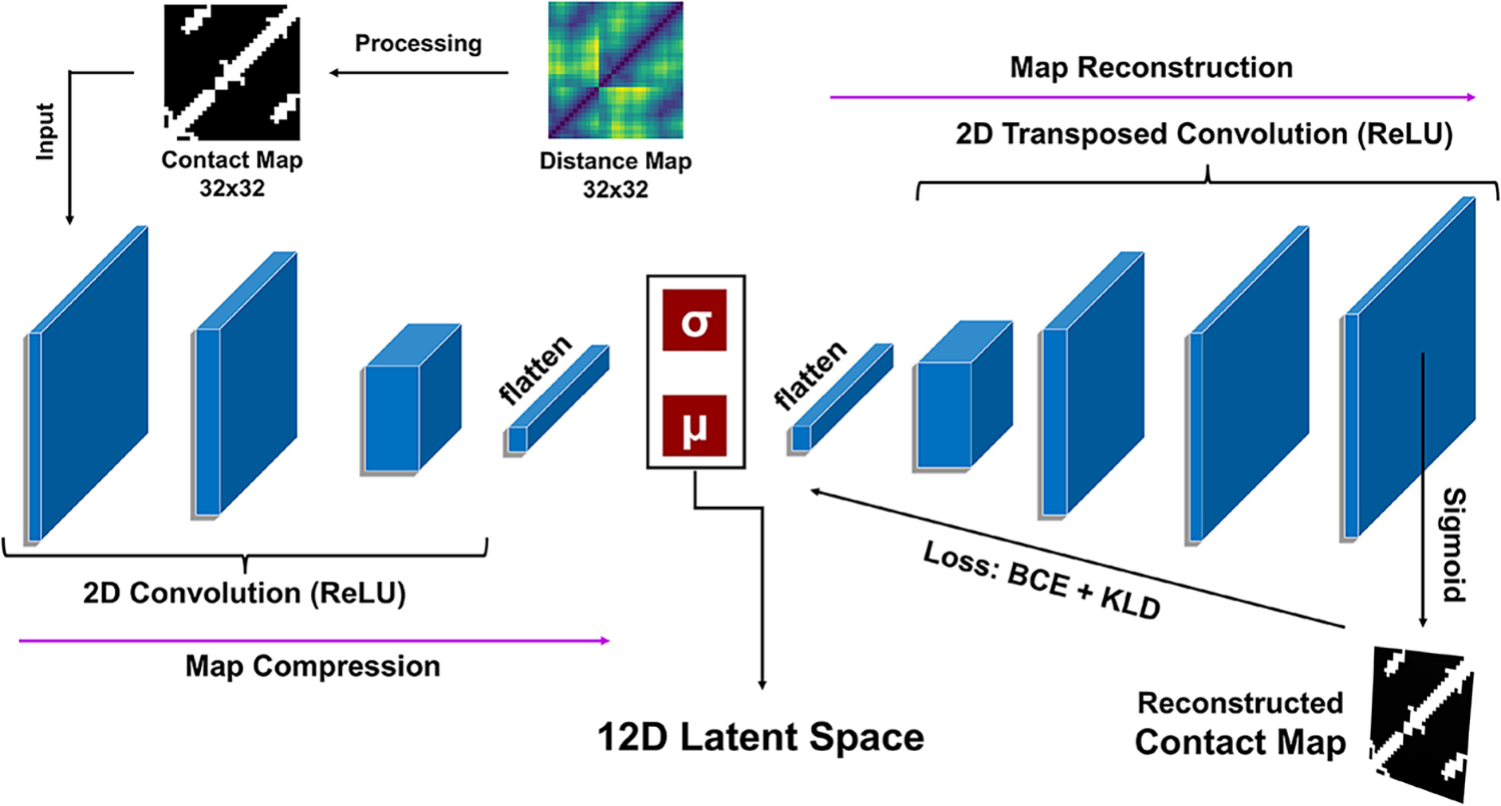
2D CVAE architecture, in which the constructed MALT1 catalytic site loop contact maps feed the ANN, compressing it through three 2D convolutional layers until generating a 12D latent space, and from this space, decompressed through four 2D convolutional layers, reconstructing the contact maps. Both compression and decompression used the ReLU activation function, and the last layer of the decoder used the sigmoid activation function for generating the reconstructed contact map. For the training procedure, binary cross-entropy (BCE) plus Kullback–Leibler divergence (KLD) function was used as a loss function.

**Table 1:** Hyperparameters Used in the Constructed 2D CVAE (Figure 2) for the Protein Environment Systems of Both Compound **1** and Compound **2**

layer	Kernel size	number of channels	stride	padding
Conv1	[4,4]	[1,16]	2	0
Conv2	[4,4]	[16,32]	2	0
Conv3	[4,4]	[32,64]	2	0
μ and σ:	flatten output from Conv3, compression from 256D → 12D			
Linear1:	decompression from 12D → 256D, reshape to feed 2D transposed convolution			
TransposeConv4	[4,4]	[64,32]	2	0
TransposeConv5	[4,4]	[32,16]	2	0
TransposeConv6	[4,4]	[16,1]	2	0
TransposeConv7	[3,3]	[1,1]	1	0

Once the 2D CVAE was correctly trained, the samples could be represented in a much more compressed space with 12 dimensions. However, it is still difficult to differentiate and analyze metastable states with this number of dimensions. Therefore, with the 12 latent variables assigned to each sample, the Pairwise Controlled Manifold Approximation (PaCMAP) algorithm was applied and could be used to reduce it from 12D to only 2D.^45,71,72^ In 2D, a much more facilitated interpretation can be obtained, and metastable state differentiation becomes clearer. In order to perform PaCMAP compression of the 2D CVAE latent space, the pacmap 0.8.0 ^71,72^ library was used. Now, for cluster search and data labeling according to metastable states, the HDBSCAN 0.8.40 algorithm^73^ was used, setting the minimum cluster size to 2000 samples and using Euclidean distance as a metric. The compression through the 2D CVAE and PaCMAP usage will be called the CVAE/PaCMAP procedure throughout the entire paper for simplicity.

### Covalent Docking for Inhibition Evaluation

With the obtained conformational landscape, the accessed metastable states from the biased MD could be clustered and labeled. Hence, from these clusters, representative MALT1 structures of each metastable state were collected, in which the conformation closest to the centroid of the cluster was chosen as representative. Once the structures were collected, covalent docking with the substrate mimic Z-VRPR-fmk could be performed to evaluate CYS472 availability, with this being an interesting strategy once the substrate mimic binds to the sulfhydryl group of CYS472; therefore, if a high energy is obtained, it shows the unavailability of CYS472 for substrate binding.^23^

For the covalent docking, AutoDockFR (ADFR)^74^ was used, and the Z-VRPR-fmk structure was collected from PDB:3 V4L,^49^ being it positioned in the obtained representative conformations by aligning the structures with the crystal. After positioning, the covalent bond between the substrate mimic and the sulfhydryl group of CYS472 could be constructed. The atoms involved in the chemical bond are the C1 atom of Z-VRPR-fmk and SG from CYS472 of MALT1, using the CB atom of CYS472 as an anchor. Now, the constructed grid box used SG coordinates for the center and dimensions (*x*,*y*,*z*) of (30 Å, 30 Å, 30 Å). After covalent docking performance, the energy of the lowest-energy pose was considered the energy of the best-oriented pose.

## Results and Discussion

In principle, compound **1** was first proposed as a MALT1 theranostic agent due to two main characteristics: it is a phenothiazine derivative, and it presents a benzothiazole portion, which provides this molecule a probe capacity through the performance of ESIPT phenomena. Compound **1**’s original work shows that this compound was designed to be a probe only;^50^ however, since it is a phenothiazine derivative, and phenothiazine derivatives were reported to have promising activity as MALT1 allosteric inhibitors,^29,75^ its choice as a theranostic agent should be a promising choice. In addition, compound **1**’s phenothiazine portion presents a very similar structure to promazine, an already reported potential MALT1 allosteric inhibitor.^29^ Nonetheless, the compound **1** phenothiazine portion is not identical to the promazine structure, and it was never tested as an MALT1 allosteric inhibitor; thus, its inhibitory performance is unknown.

In this context, this motivated a modification in the phenothiazine portion of compound **1**, generating compound **2**. Compound **2** presents the same benzothiazole portion as compound **1**; however, it presents a different phenothiazine portion, being identical to the thioridazine portion, a reported promising MALT1 allosteric inhibitor, with slightly better inhibition than promazine.^29^ Therefore, even based on the fact that compound **1**’s inhibitory assessment is unknown, it is expected that compound **2** will present a better inhibitory capacity, once its phenothiazine portion is identical to thioridazine, while compound **1**’s phenothiazine portion is not identical to promazine, only very similar. That said, the formal analysis for both compounds is presented below in a comparative way, taking into consideration the expectations presented above.

### Docking of Compounds **1** and **2** for the Initial Structure of the Protein–Ligand Complex

The obtained initial structure of the protein–ligand complex for compound **1** in protein environment simulations showed that the best-oriented pose presented a docking energy of −8.3 kcal/mol. The ligand mainly presented pi-alkyl and alkyl interactions with the protein surroundings, the surroundings being the amino acid residues LEU354, VAL389, ALA402, and PHE406 (Figure S4). In addition, no hydrogen-bonding interaction was observed between the −OH group of the ligand and surrounding amino acid residues, showing the hydrophobicity of the MALT1 allosteric pocket.

Regarding compound **2**, as an initial structure for the protein environment simulations, the best pose orientation presented an energy of −6.7 kcal/mol. Following the same procedure as obtained for compound **1**’s initial structure, compound **2** mainly interacts with the protein surroundings by pi-alkyl and alkyl interactions; however, new interacting amino acid residues were observed. It was observed that there were interactions between compound **2** and amino acid residues VAL352, LEU354, LYS387, LEU391, LEU409, TRP588, and ALA591 (see Figure S10). Just as observed for compound **1**’s initial structure, no hydrogen bond interaction was observed between the −OH group and the amino acid residues in the surroundings, reinforcing the hydrophobicity of the MALT1 allosteric pocket. Therefore, once the initial protein–ligand complex structure for both compounds was determined, the simulations in the protein environment could be performed and further analyzed.

### ESIPT On/Off Distribution

Starting with ESIPT on/off distribution assessment, Figures 3 and 4 show the distribution of compounds **1** and **2**, respectively, in the simulated environments. Furthermore, the convergence of the dihedral angle distributions was assessed for all systems (Figure S3). The remarkable consistency observed over the course of the simulations indicates that all of the systems attained a stationary regime.

Hence, Compound **1**’s population distribution was first evaluated in both water-only and protein environments. Figure 3A shows the dihedrals 1 and 2 (i and ii, respectively), and Figure 3B,C shows their distribution in water and protein environments, respectively. From this evaluation, it is possible to observe the ESIPT on/off ratio.^40^ It is important to mention that according to the Eigen-Weller model,^62,63^ ESIPT only occurs if there is an intramolecular interaction of hydrogen bonds between the donor (−OH) and acceptor (−N =) groups, which is called ESIPT on. If this interaction ceases, then no ESIPT occurs, making it an ESIPT off. Thus, this model was used to define whether an accessed metastable state presented an ESIPT on or off.

**Figure 3:**
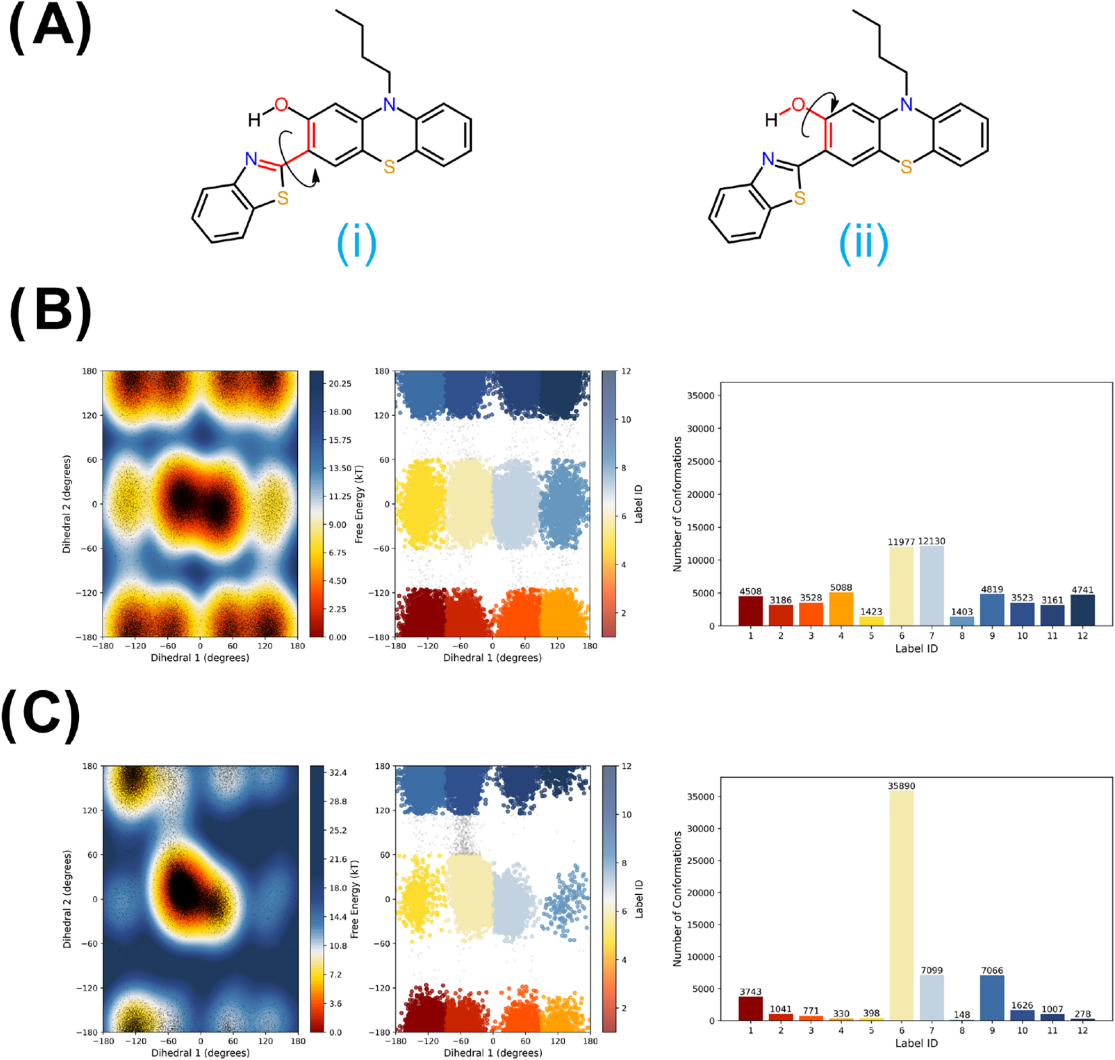
(A) Structure of compound **1** and calculated dihedrals, being (i) dihedral 1 and (ii) dihedral 2, two important dihedrals to evaluate the intramolecular interaction of hydrogen bonds between the donor (−OH) and acceptor (−N =) groups. (B, C) Dihedrals’ distribution in water and protein environments, respectively, with the associated labeling of the accessed metastable states and their population distribution as a bar plot.

For compound **1** in the water-only environment (Figure 3B), the two most populated metastates were metas 6 and 7, with populations of 11977 and 12130, respectively. The same occurred in the protein environment (Figure 3C), with the most populated metas also metas 6 and 7, with populations of 35890 and 7099, respectively. Metas 6 and 7, in both environments, are represented by conformations with almost no dihedral 1 and 2 fluctuation, enabling these conformations to preserve the intramolecular interaction of hydrogen bonds between the donor (−OH) and acceptor (−N =) groups. In fact, in both environments, the average distance between OH···N of metas 6 and 7 is kept below 2.20 Å, while for other metas, it is higher than 3.50 Å, a distance that prevents the occurrence of such an intramolecular interaction (see Table S4 for calculated data for each meta). In this sense, metas 6 and 7 are the only ones with appropriate geometry capable of performing the ESIPT reaction. Therefore, metas 6 and 7 represent ESIPT on conformations, and the others, ESIPT off conformations.

In that regard, the ESIPT on/off ratio can be obtained from the population ratio of the described metas. In this sense, for the water-only environment, the on/off ratio was 40/60%, and for the protein environment, 72/28%. If the angle distributions for the ESIPT on conformations are considered, Figure S7 shows a negligible deviation when compared to only using the distance criteria, reinforcing the mentioned ratios. Therefore, there is a substantial increase in ESIPT on conformations in the protein environment when compared to the water-only environment, mainly due to the hydrophobicity of the MALT1 allosteric pocket, which poorly stabilizes dihedrals 1 and 2 fluctuations, implying an enhanced keto emission for compound **1** when signaling the MALT1 target. In the water-only environment, a higher enol emission is expected, as this environment is capable of stabilizing ESIPT off conformations by forming intermolecular hydrogen bonds between water molecules and both donor and acceptor groups, presenting an enhanced dihedral 1 and 2 fluctuation.

Now, regarding compound **2**’s ESIPT on/off distribution, from Figure 4A, it is possible to observe compound **2**’s structure and the calculated dihedrals for ESIPT on/off evaluation. Figure 4B,C shows the dihedrals’ distribution in water-only and protein environments, respectively. Similarly, from what was observed in the accessed compound **1** distribution, the observed distributions generated by using compound **2** show a more uniform distribution in the water-only environment (Figure 4B), while showing a higher number of conformations around dihedrals 1 and 2 equal to 0 in the protein environment (Figure 4C). Thus, metas 6 and 7 are considered ESIPT on conformations due to the maintenance of the intramolecular interaction of hydrogen bonds between the donor (−OH) and acceptor (−N =) groups, with average distances below 2.20 Å (see Table S5 for the calculated data for all groups).

**Figure 4:**
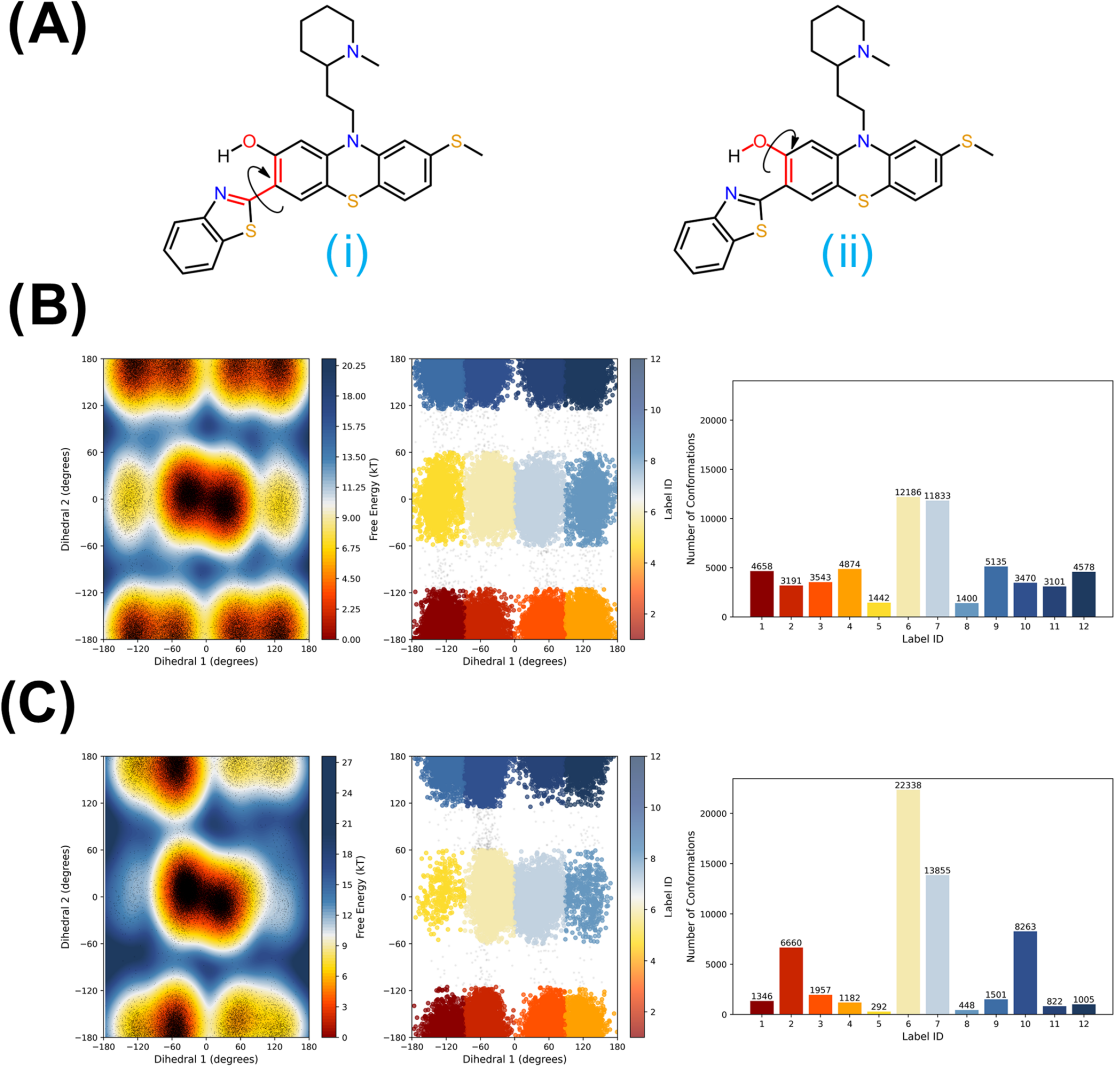
(A) Structure of compound **2** and the calculated dihedrals, being (i) dihedral 1 and (ii) dihedral 2, two important dihedrals to evaluate the intramolecular interaction of hydrogen bonds between the donor (−OH) and acceptor (−N =) groups. (B, C) Dihedrals’ distribution in water and protein environments, respectively, with the associated labeling of the accessed metastable states and their population distribution as a barplot.

Considering metas 6 and 7, it was observed that in the water-only environment, there were 24019 conformations, and in the protein environment, 36193, an increase of 51%, mainly due to meta 6, whose population almost doubled compared to the water environment distribution in the protein environment. In addition, it is important to mention that when comparing the distribution in the protein environment generated by compounds 1 and 2 (compare Figures 3C and 4C), a slight increase in the population of metas located around dihedral 2 equals −180 and 180° was observed. This observation indicates a higher interaction between compound **2** and the amino acid residues capable of stabilizing the dihedrals’ fluctuations, which enabled more conformations to be stabilized through intermolecular interactions of hydrogen bonds between these amino acid residues and donor and acceptor groups of compound **2**.

In that regard, the ESIPT on/off ratio for compound **2** can then be obtained through population analysis. In the water environment, an ESIPT on/off ratio of 40/60% was observed, and in the protein environment, 60/40%. If the angle distributions for the ESIPT on conformations are considered, Figure S13 shows a negligible deviation when compared to only using the distance criteria, reinforcing the mentioned ratios. Therefore, a substantial difference was observed in the ESIPT on/off ratio of compound **2** when comparing the environments. From this, a 51% increase in ESIPT on conformations from the water-only environment to the protein environment was observed, followed also by a 51% decrease in ESIPT off conformations. However, when comparing compound **1** and **2** ratios in the protein environment, a higher fraction of ESIPT off conformations is observed for compound **2**. Therefore, both compounds showed a trend to increase their keto emission population in the protein environment but with a slightly higher enol emission population for compound **2**.

### ESIPT Spectroscopy

Moving further, representative structures were collected from the metastable states. Therefore, as representative conformations, the closest conformation of the centroid of the observed metastable state cluster was selected, following the previously described procedure,^23,40^ totaling 12 conformations for each system of both simulated compounds. Starting with compound **1**, its representative conformations with the chemical environment were submitted to quantum TDDFT calculations, and the obtained spectroscopic values are reported in Table 2.

**Table 2:** Calculated Emission Wavelengths with TDDFT of the Collected Representative Conformations of the Accessed and Labeled Metastable States of Compound **1**

	water env. emission (nm)		protein env. emission (nm)	
meta ID	enol	keto	enol	keto
1	509.79	–	461.05	–
2	497.30	–	449.74	–
3	495.78	–	449.97	–
4	510.17	–	459.31	–
5	521.48	–	467.02	–
6	–	556.67	–	556.94
7	–	555.96	–	556.92
8	521.72	–	471.78	–
9	510.16	–	459.90	–
10	500.08	–	449.71	–
11	497.10	–	446.55	–
12	510.47	–	459.26	–
average	507.40	556.31	453.50	556.93

It is possible to observe from Table 2 that for both environments, the compound **1** keto emission occurred in the green region, with an average wavelength emission of 556.31 nm for the water environment and an average wavelength emission of 556.93 nm for the protein environment, both with negligible standard deviation between the obtained values. Now, regarding the enol emission, a difference is observed between environments. In the water environment, an average wavelength emission of 507.40 nm with a standard deviation of 9.11 nm between the obtained values was observed, while in the protein environment, an average wavelength emission of 453.50 nm and a standard deviation of 7.85 nm between the obtained values were observed. Therefore, it was possible to observe that in the water environment, the enol emission occurred in the cyan region and in the protein environment, in the blue region. In addition, a Stokes shift of 48.91 and 103.43 nm was observed for the water-only environment and protein environment, respectively, enabling the enol emission in a different region of the visible light spectrum when signaling the MALT1 protein, showing that for the proposed probe, the enol emission is of utmost importance, being this emission capable of differentiating environments and acting as a fingerprint. These results demonstrate the significant role of enol emission in the evaluation of ESIPT-based probes for biological target signaling, which is a topic that has received limited attention in previous studies.

Now, regarding compound **2**, calculations with the collected representative conformations, it is possible to observe the spectroscopic data obtained for this compound reported in Table 3. From Table 3, it is possible to observe the same behavior as that observed for compound **1** (Table 2): a cyan enol emission in the water-only environment, a blue enol emission in the protein environment, and a green keto emission for both environments. In the water environment, an average enol emission of 505.68 nm with a standard deviation of 7.96 nm between the obtained values was observed, and an average keto emission of 556.62 nm with a negligible standard deviation between the obtained values was observed. Now, for the protein environment, an average enol emission of 454.90 nm with 8.27 nm of standard deviation between the obtained values was observed, followed by an average keto emission of 556.78 with a negligible standard deviation between the obtained values. For the environments, Stokes shifts of 50.94 and 101.88 nm for the water-only environment and protein environment, respectively, were observed. Therefore, the obtained value for compound **2** is similar to what was observed for compound **1**, reinforcing the major importance of enol emission of the proposed probes when signaling the MALT1 protein.

**Table 3:** Calculated Emission Wavelengths with TDDFT of the Collected Representative Conformations of the Accessed and Labeled Metastable States of Compound **2**

	water env. emission (nm)		protein env. emission (nm)	
meta ID	enol	keto	enol	keto
1	508.69	–	459.30	–
2	494.36	–	448.86	–
3	496.73	–	447.23	–
4	508.69	–	458.73	–
5	517.49	–	466.56	–
6	–	556.46	–	556.78
7	–	556.78	–	556.77
8	516.58	–	471.88	–
9	509.78	–	459.36	–
10	498.98	–	448.92	–
11	496.81	–	445.80	–
12	508.69	–	459.37	–
Average	505.68	556.62	454.90	556.78

The evaluation of both compounds showed an interesting result: the enol emission is the crucial feature for differentiating the environments and making an appropriate signaling of the MALT1 protein once it occurs in different regions of the visible light spectrum. In contrast, keto emission did not help to differentiate environments once it occurred in the same region in both environments. In this sense, the ESIPT performance does not favor the signaling purposes of the investigated compounds; however, the ESIPT-based portion of the structures guarantees different enol fluorescence for the compounds when signaling different chemical media. Thus, different from what is majorly explored in the literature regarding ESIPT-based chemical probes for biological targets, which mainly focus on keto emission, the results presented so far show that when signaling a biological target, enol emission can be crucial to differentiate chemical media and image the chosen target. This implies that an evaluation based solely on keto emission might hide crucial features when aiming at diagnostic agents, making it very important to also evaluate enol emission.

Considering the critical role of enol emission in the proposed MALT1 signaling mechanism, the fraction of ESIPT off conformations becomes a key parameter. In general, a higher population of enol conformations in the protein environment is expected to increase the enol emission intensity, thereby enhancing the signaling response of the MALT1 system. On the basis of the calculated ESIPT on/off ratios, both compounds exhibit significant populations of enol conformations in the protein environment, supporting their potential use as MALT1 signaling probes. However, compound **2** demonstrates superior performance, presenting an enol emission contribution of 40% compared with 28% for compound **1**. This higher population of enol conformations for compound **2** is expected to produce a stronger enol emission signal upon interaction with MALT1, indicating that compound **2** represents the more suitable probe for signaling applications (see Figures S3 and S7 for emission population comparisons).

In this regard, the results obtained thus far establish both compounds as promising modulators of MALT1 signaling. The ESIPT emission spectrum, considering the water-only medium and the protein environment, presents a substantial difference, in which water enol emission (cyan) occurs in a different region of the protein enol emission (blue), as shown in the scheme presented in Figure 5. In addition, the performance of full QM calculations for a few conformations shows the same emission regions regarding the enol and keto emissions in different environments (Table S6), supporting the presented results using the ONIOM procedure. Therefore, the presence of blue emission when signaling MALT1 protein acts as a fingerprint for the comprehension of what is being signaled, an interesting advance in ESIPT scientific literature, which often focuses on keto emission rather than enol emission for signaling purposes. Therefore, it is possible to move further and evaluate compounds **1** and **2**’s inhibitory capacity through allosteric positioning in MALT1, aiming for a compound not only for cancer diagnosis but also for theranostics purposes.

**Figure 5:**
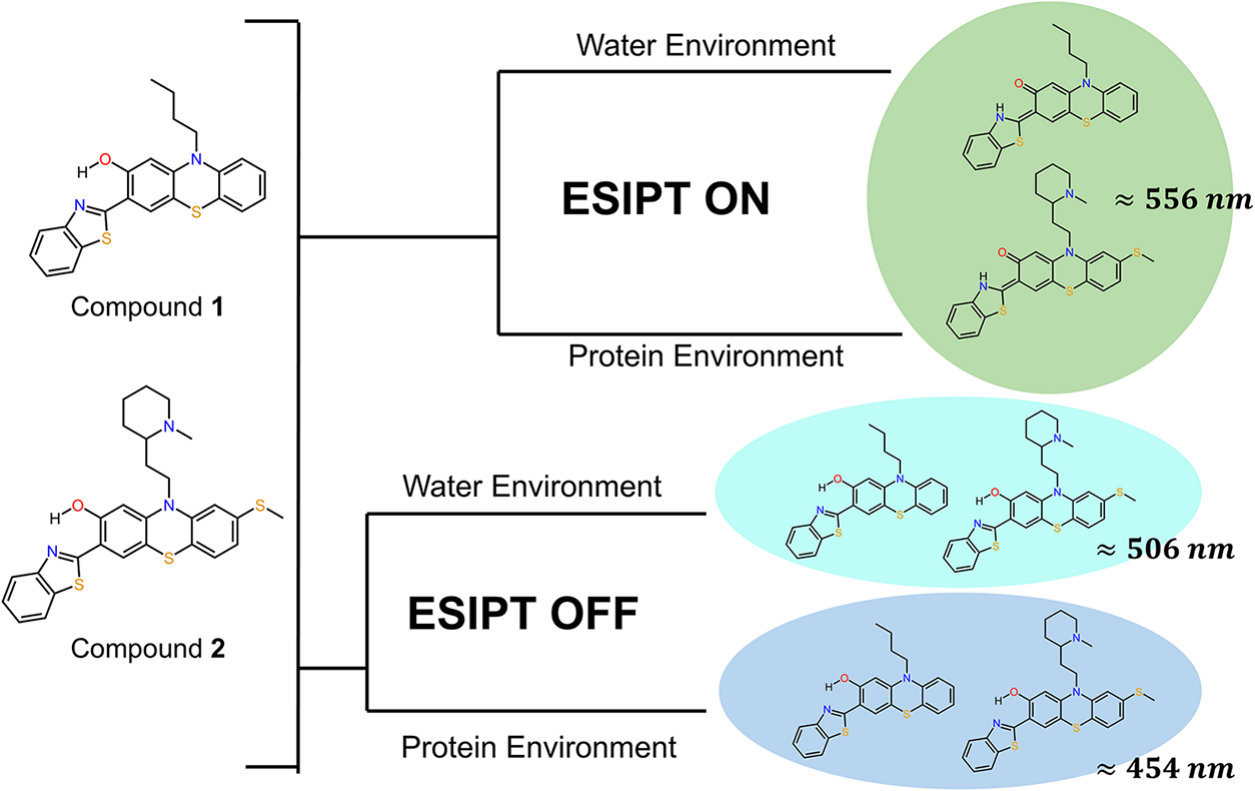
Scheme of the obtained TDDFT emission data considering the environments. Both compounds showed a green ESIPT on emission in both water and protein environments, a cyan enol emission in the water environment, and a blue enol emission in the protein environment. From this, the protein signaling could be differentiated through enol emission rather than keto emission.

### Catalytic Site Loops Distribution and Inhibitory Insights

Aiming at theranostic purposes, the catalytic site loop (loop 1 and loop 3) distribution was investigated in order to observe patterns stabilized in metastable states. From this, it would be possible to obtain information regarding inhibition, representative conformations for substrate mimic docking, and the population distribution of the accessed metastable states from biased MD production. In this sense, starting with the compound **1** protein environment system, Figure 6 shows the obtained latent space from CVAE/PaCMAP (see Figure S6 for the model training profile) with the obtained representative structures of each accessed metastable state, highlighting loops 1 and 3 conformations.

**Figure 6:**
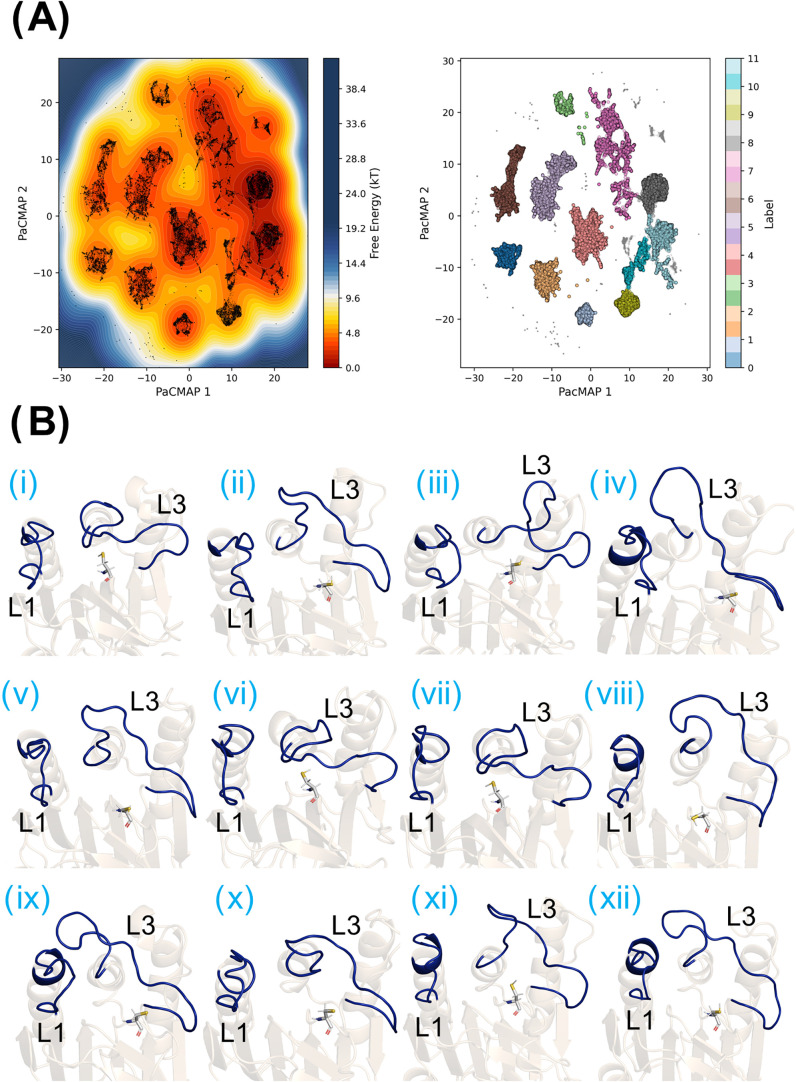
Metastable states distribution obtained from CVAE/PaCMAP compression of compound **1** allosterically positioned in MALT1, being (A) the obtained latent space with the associated cluster labels and (B) the representative structures of each metastable state, going from i to xii, representing the labeled groups from 0 to 11, respectively. In (B), loops 1 and 3 are highlighted in blue and annotated, and the catalytic cysteine is shown as sticks.

For the built model for compound **1**, with the 10 samples separated for external validation, Table 4 shows the metrics calculated per separate sample, namely, *R* ^2^, MSE, MAE, and SSIM. In this sense, map reconstruction achieved an overall Structure Similarity Index Measure (SSIM) of 0.9603 ± 0.0135, showing an excellent similarity between the true map and the reconstructed one. The high similarity between the true maps and their reconstruction shows that the built model presents a very accurate latent representation of the accessed conformations. To observe the comparison between the true maps and their reconstruction, see Figure S8.

**Table 4:** Calculated Metrics for the Trained CVAE Model for Compound **1** in the Protein System Using the 10 Samples Separated for External Validation

sample #	*R* ^2^	MSE	MAE	SSIM
1	0.8853	0.019086	0.036481	0.9398
2	0.9236	0.012895	0.027214	0.9600
3	0.9480	0.009001	0.022295	0.9731
4	0.9466	0.007663	0.018129	0.9723
5	0.9256	0.011054	0.022004	0.9612
6	0.9463	0.008252	0.018733	0.9723
7	0.9541	0.006702	0.018123	0.9759
8	0.9028	0.014812	0.027926	0.9492
9	0.8798	0.018598	0.032975	0.9364
10	0.9288	0.011097	0.028482	0.9626
average	0.9241 ± 0.0253	0.011916 ± 0.004172	0.025236 ± 0.006087	0.9603 ± 0.0135

Now, regarding the sensitivity of PACMAP clustering to different seeds, three different seeds, namely, 13, 42, and 109, were compared to the used PACMAP space for analysis (original) through procrustes analysis.^76^ The obtained raw PACMAP spaces for each tested seed and the original can be seen in Figure S9A. From the analysis for the model of compound **1**, an overall disparity of 0.06955 ± 0.00952 was obtained, showing that different seeds present a nearly perfect similarity with the original PACMAP space used for analysis. In addition, Figure S9B shows the alignment between the tested PACMAP space seeds and the original PACMAP space, where it is possible to observe that different seeds present the same clusters. Therefore, compound **1** model presents a negligible sensitivity to different seeds, implying great robustness and systematic reproducibility of the conformational landscape.

From the performed analysis, it was observed that by positioning compound **1** in the MALT1 allosteric pocket, a high conformational diversity for loops 1 and 3 was observed, in which 12 metastable states were stabilized, as shown in Figure 7. In the observed distribution, the most populated metastate presented 8266 structures, being it meta 7 (Figure 6B viii), and the lowest populated metastate, 2234 structures, being it meta 3 (Figure 6B iv). Therefore, no very large groups were observed in which the most populated meta represented only 14.9% of the labeled data. The observations imply that positioning compound **1** in the allosteric pocket of MALT1 allows high degrees of freedom for catalytic site loops 1 and 3.

**Figure 7:**
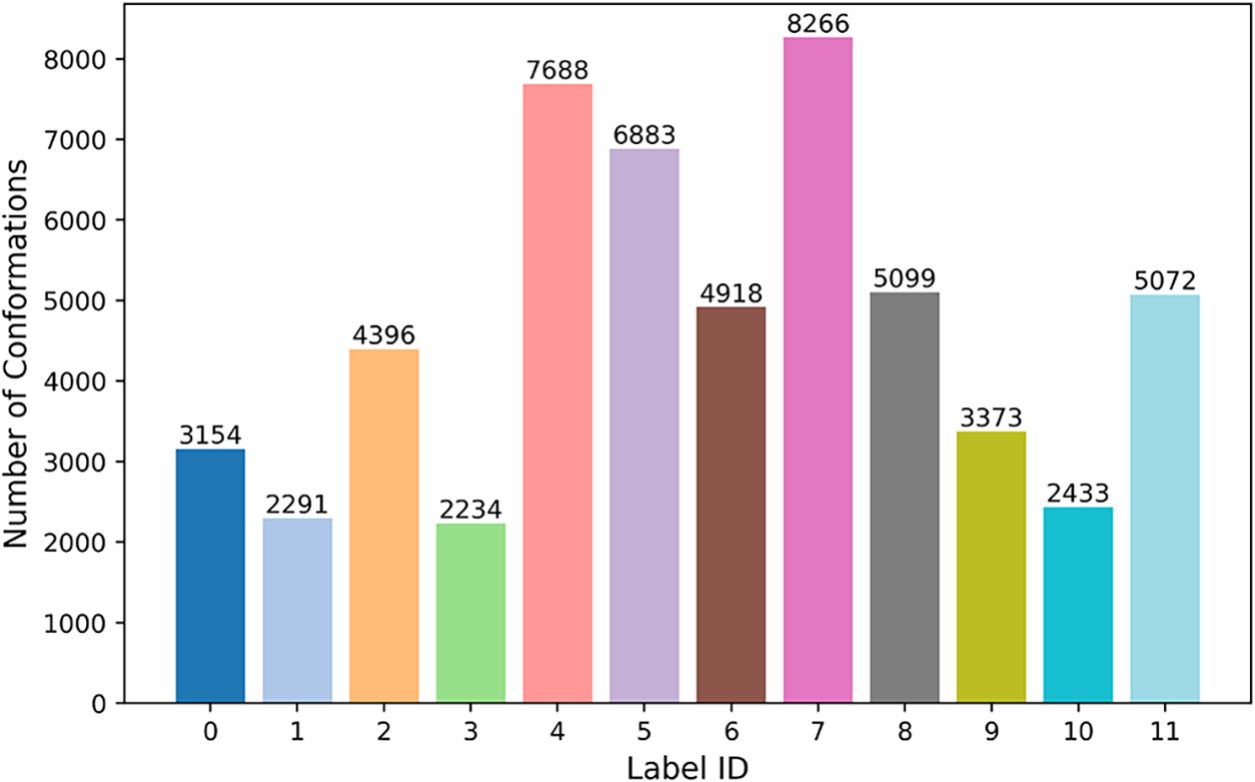
Population distribution of labeled metastable states, shown in Figure 6A, observed from the CVAE/PaCMAP compression latent space.

Regarding the obtained representative structures for each accessed metastable state, it was observed that only a small portion presented loop 1 and 3 conformations more likely to make CYS472 unavailable for substrate binding, which is crucial for MALT1 inhibition. Metas 2, 5, and 6 (Figure 6B; iii, vi, and vii, respectively) showed aggressive loop 1 and 3 bending toward CYS472, covering this important catalytic residue and making it more unavailable for substrate binding. The three mentioned metas together represent 29% of the labeled data, being intermediary populated metas, in which metas 2, 5, and 6 presented populations of 4396, 6883, and 4918 structures, respectively. Therefore, 71% of the labeled data presented loops 1 and 3 conformations that were less bent toward the catalytic site, generating structures with a much more available and uncovered catalytic cysteine.

In addition, free energies were calculated for each labeled metastable cluster and can be seen in the bar plot in Figure 8B together with the computed 3D free energy surface onto PACMAP space (Figure 8A). The most stable state, being meta 7, was set to 0 energy, presenting an energy difference of 2.39 kT from the less stable state, meta 9. Furthermore, metas 11, 8, and 4 basins presented close energies to meta 7, with differences of 0.36, 0.38, and 0.55 kT, respectively, showing similar stability.

**Figure 8:**
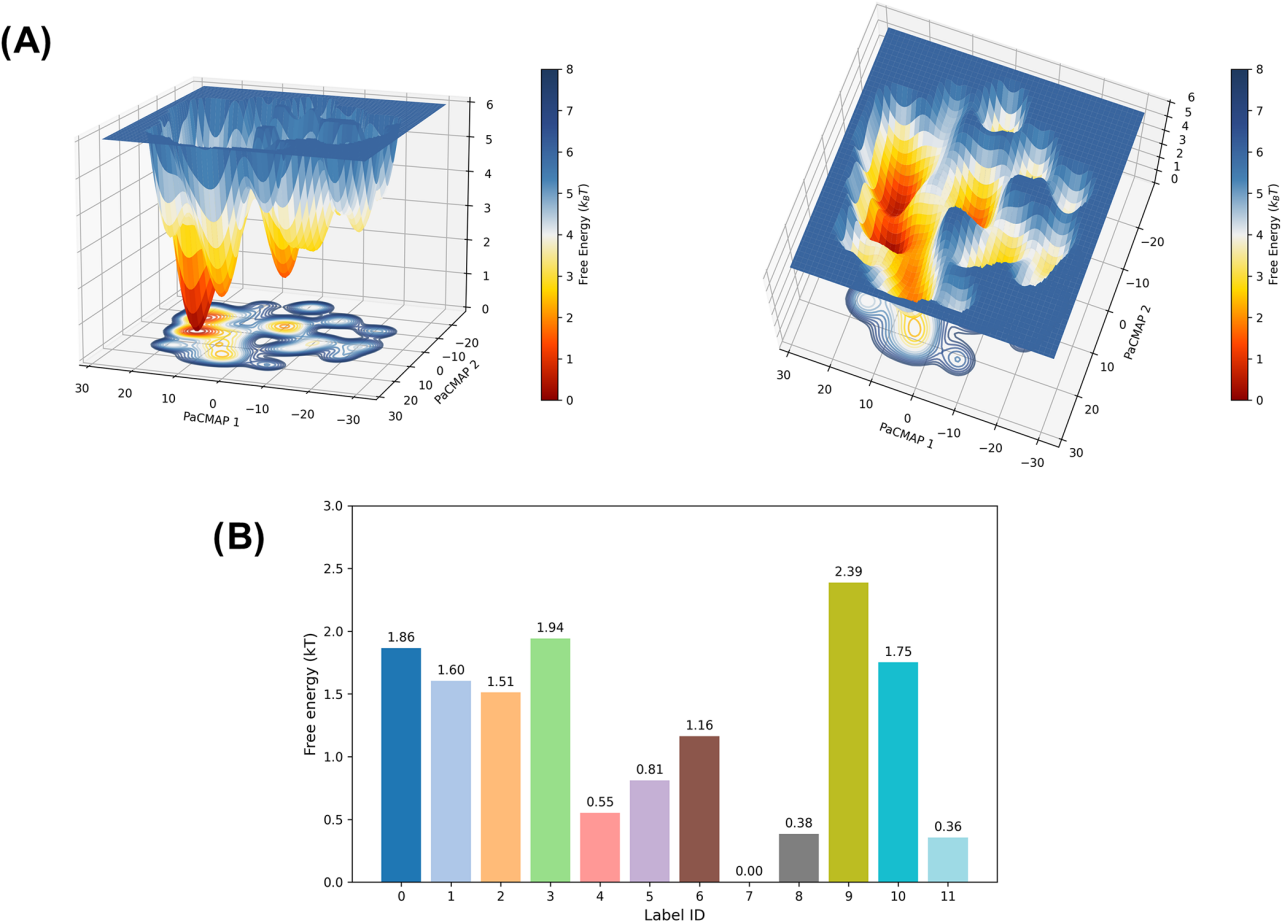
(A) 3D free energy surface onto the obtained PACMAP space of compound **1** CVAE model, shown in the horizontal and top perspective. (B) Computed free energies of the labeled metastable state basins.

Now, regarding compound **2**, to evaluate the inhibitory capacity of compound **2**, the catalytic site loop distribution of MALT1 when compound **2** is positioned in its allosteric pocket was evaluated through the CVAE/PaCMAP procedure (see Figure S12 for model training profile), and the obtained distribution is presented in Figure 9A. In addition, Figure 9B presents the representative conformations of each labeled metastable state observed in the distribution.

**Figure 9:**
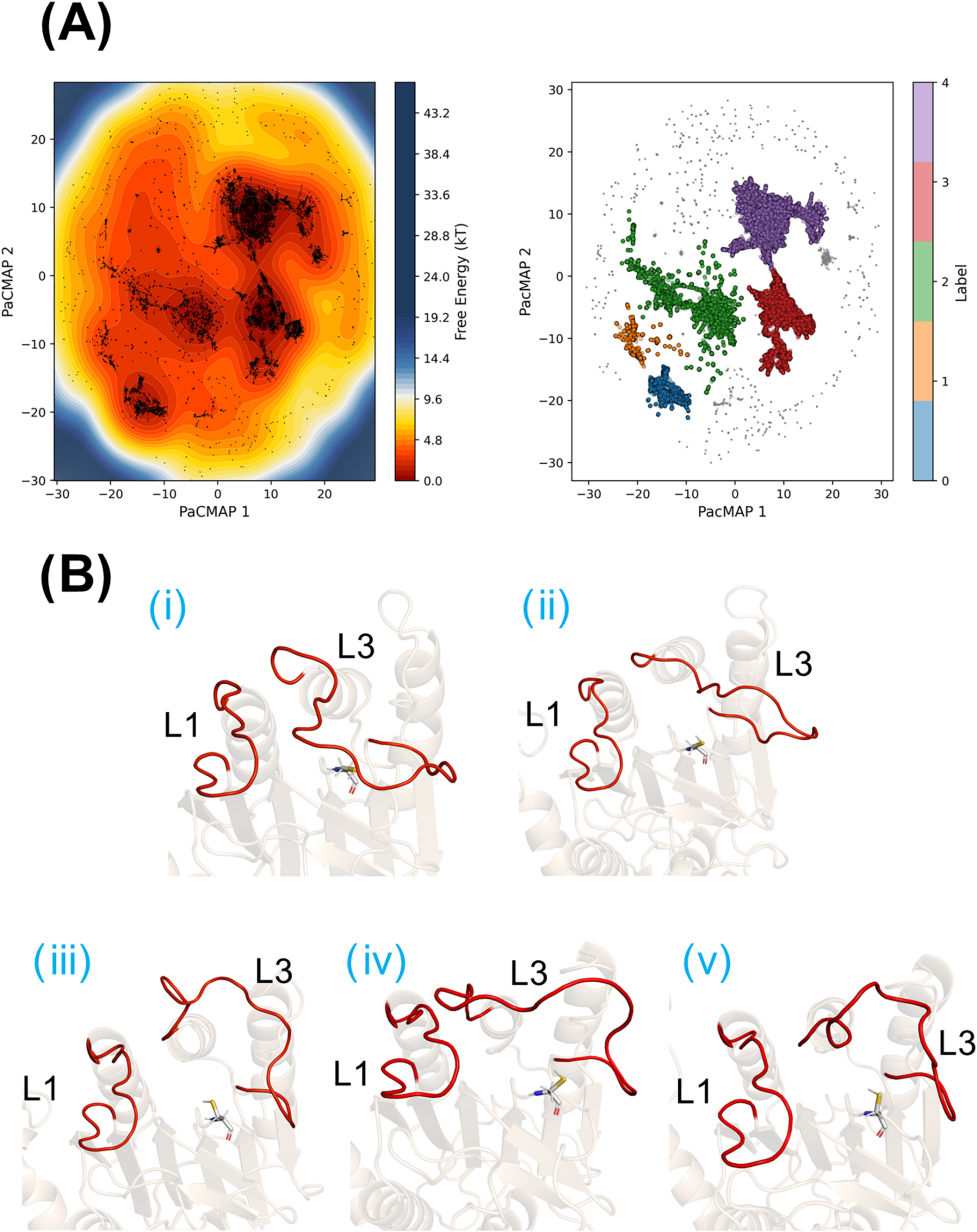
Metastable states distribution obtained from CVAE/PaCMAP compression of compound **2** allosterically positioned in MALT1, being (A) the obtained latent space with the associated cluster labels and (B) the representative structures of each metastable state, going from i to v, representing the labeled groups from 0 to 4, respectively. In (B), loops 1 and 3 are highlighted in red and annotated, and the catalytic cysteine is shown as sticks.

For the model built using compound **2**, Table 5 shows the calculated *R* ^2^, MSE, MAE, and SSIM for each one of the 10 samples separated for external validation. Therefore, for the compound **2** model, an overall SSIM of 0.9876 ± 0.0070 was obtained, showing a very high similarity between the true maps and its reconstruction. This implies that the built model presents a very accurate latent representation of the accessed conformations. Therefore, for observing the comparison between the true maps and their reconstruction, see Figure S14.

**Table 5:** Calculated Metrics for the Trained CVAE Model for Compound **2** in the Protein System Using the 10 Samples Separated for External Validation

sample #	*R* ^2^	MSE	MAE	SSIM
1	0.9977	0.000314	0.002661	0.9988
2	0.9737	0.003637	0.005935	0.9865
3	0.9516	0.007008	0.009799	0.9757
4	0.9881	0.001889	0.005518	0.9940
5	0.9777	0.003511	0.008893	0.9886
6	0.9820	0.002467	0.004855	0.9910
7	0.9521	0.007760	0.015413	0.9755
8	0.9693	0.004930	0.009262	0.9845
9	0.9806	0.003044	0.005576	0.9902
10	0.9816	0.002574	0.005663	0.9907
average	0.9754 ± 0.0139	0.003713 ± 0.002167	0.007357 ± 0.003415	0.9876 ± 0.0070

Now, to evaluate the sensitivity of the PACMAP clustering to different seeds, three different seeds were tested, namely, 13, 42, and 109, in which the comparison used as reference the PACMAP space used for analysis (original). Just as performed for the compound **1** model, the compound **2** model PACMAP sensitivity was also evaluated through procrustes analysis. The obtained raw PACMAP spaces for each tested seed and the original space can be seen in Figure S15A. From the analysis for the compound **2** model, an overall disparity of 0.27603 ± 0.00509 was obtained. Despite presenting a higher overall disparity than the one obtained for the compound **1** model, Figure S15B shows through the PACMAP spaces of the different seeds aligned with the original space that the same clusters were obtained. Therefore, the higher disparity arises due to higher data sparsity, especially regarding metas 0 and 1 (lower populated metas). However, the compound **2** model still presented a systematic representation of the same clusters, showing appropriate reproducibility of the conformational landscapes.

At a first glance, and by comparing the obtained catalytic site loops distribution for compound **2** with compound **1** (compare Figures 9A and 6A), a substantial difference was noticed regarding the metastable states’ distribution: compound **2** stabilized many fewer metastable states than compound **1**. This difference causes the MALT1 metastates stabilized from usage of compound **2** to be much more populated, as can be seen in the obtained population distribution shown in Figure 10. It was observed for compound **2** that the most populated group was meta 4, with 16369 conformations, representing 36.4% of the labeled data, more than double the most populated meta observed for compound **1**, which was 14.9%. In addition, from the five accessed metastable states for compound **2**, three of them present populations higher than 10000 conformations.

**Figure 10:**
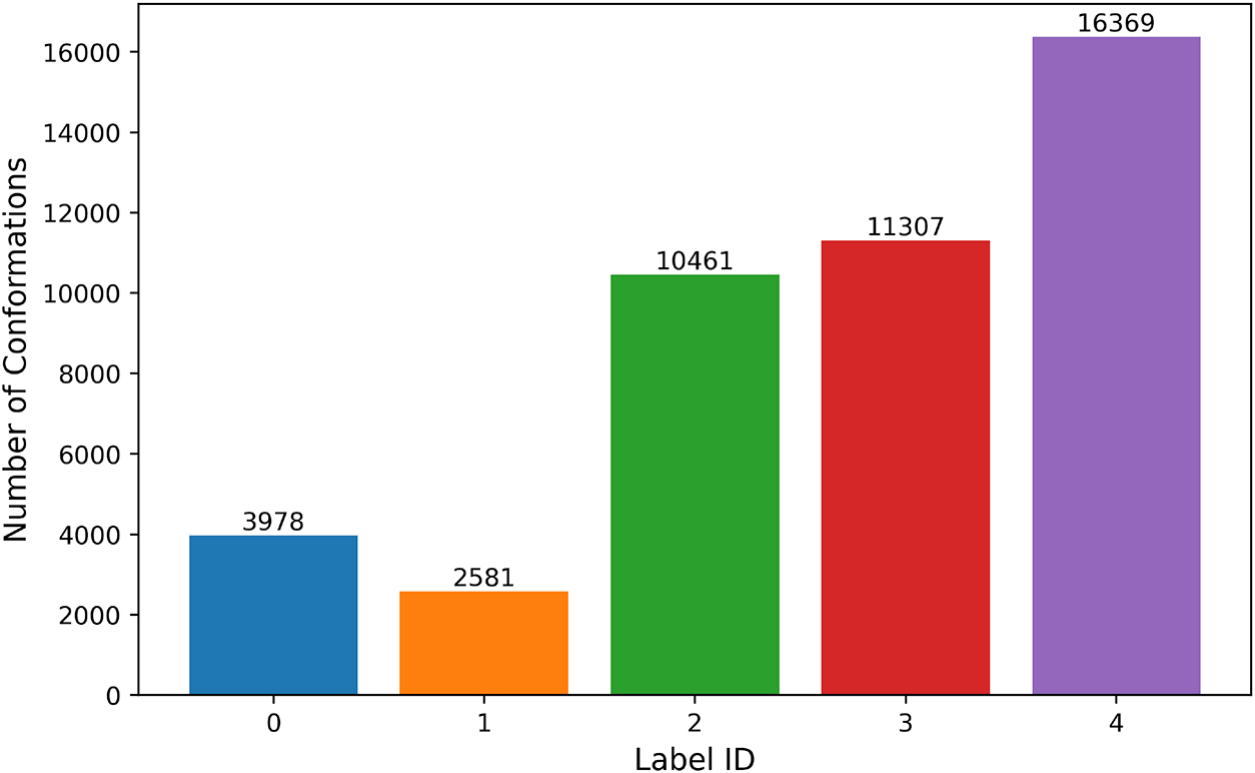
Population distribution of labeled metastable states shown in Figure 9A, observed from the CVAE/PaCMAP compression latent space.

From the observation of the representative conformations obtained for loops 1 and 3 by using compound **2**, it was noticed that three of the five observed metastates presented a more aggressive loop 1 and 3 bent toward the catalytic cysteine. The metas that showed this behavior were metas 0, 3, and 4, and the observed conformations show that these metas probably stabilized loop 1 and 3 conformations with an unavailable CYS472, a crucial feature for MALT1 inhibition. In addition, metas 0, 3, and 4 together totalize 31654 conformations, representing 71.0% of the labeled data, leaving only 29.0% of conformations with less bent loops 1 and 3 toward the catalytic cysteine, implying an available CYS472 for substrate binding. Therefore, compound **2** presented a substantial increase of stabilized conformations with loops 1 and 3 bent toward CYS472 when compared to compound **1**, showing the promising capability of compound **2** to prevent substrate binding in MALT1 and present an increased inhibitory ratio.

Figure 11B illustrates the computed free energy landscape of the metastable basins identified for compound **2**. The meta 4 state exhibited the highest thermodynamic stability and was therefore adopted as the reference state (Δ*G* = 0 kT). Interestingly, meta 2 displayed an almost identical stability, with a negligible free energy difference of only 0.02 kT, whereas meta 3 was slightly less favorable (Δ*G* = 0.24 kT). The least stable conformations corresponded to meta 0 and meta 1, with relative free energies of 1.10 and 1.46 kT, respectively, compared with meta 4.

**Figure 11:**
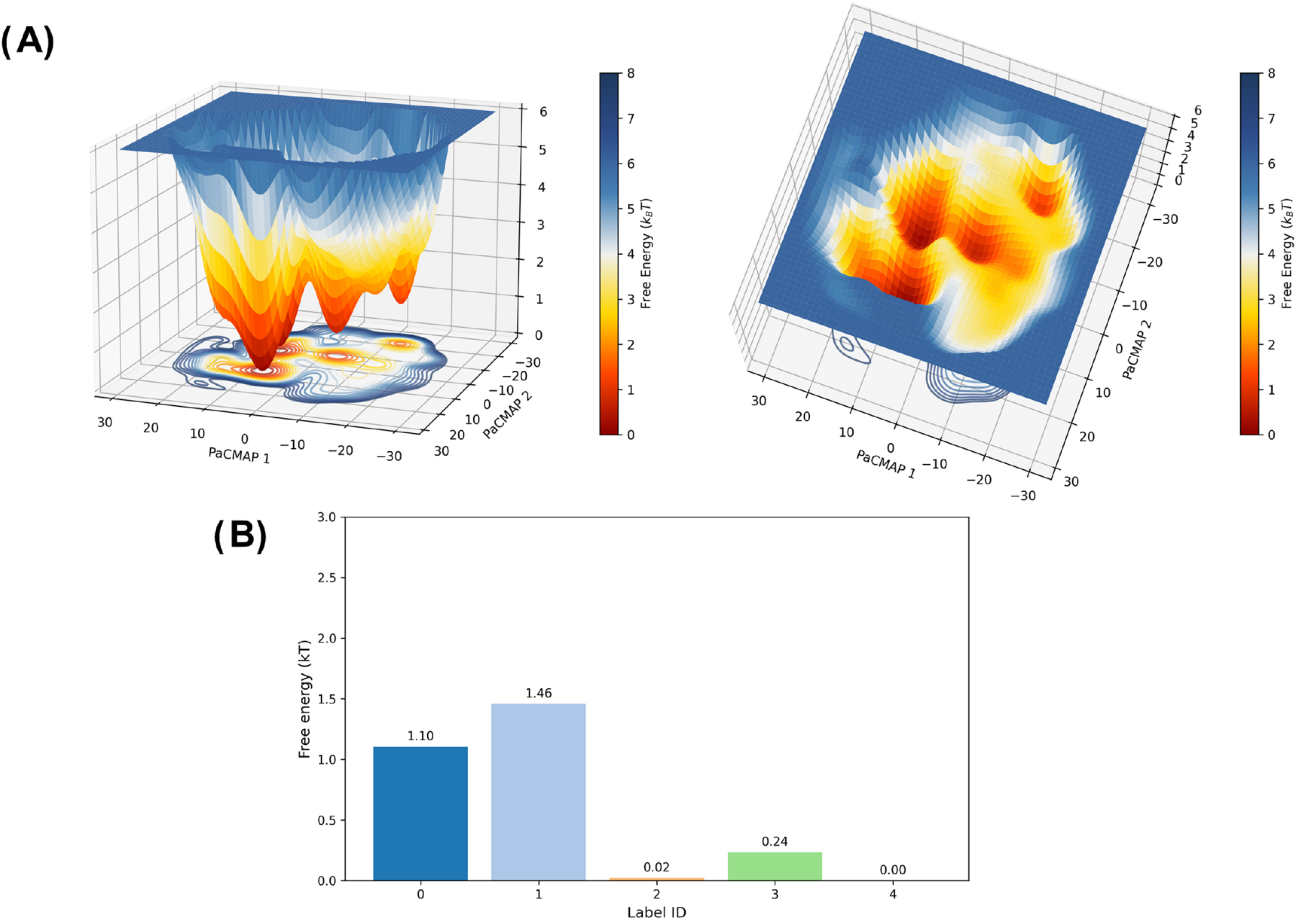
(A) 3D free energy surface onto the obtained PACMAP space of compound **2** CVAE model, shown in the horizontal and top perspective. (B) Computed free energies of the labeled metastable states basins.

In this scenario, regarding the translational comprehension of these findings to human MALT1 behavior, Figure 12 shows the structural comparison among meta 6 of compound **1** simulations, meta 4 of compound **2** simulations, and the human MALT1 crystal PDB: 6F7I^28^ allosterically inhibited with MLT-747. Both loops 1 and 3 present two mismatch residues each between 3 V4L and human MALT1, being for loop 3 ALA504-GLU505 in 3 V4L and CYS496-GLN497 for 3 V55, and for loop 1 SER360/TRP362 in 3 V4L and ASN352/ARG354 in 3 V55. This can differ the dynamical behavior of the catalytic loops; however, the effect obtained through allosteric inhibition is similar. As can be seen in Figure 12, loops 1 and 3 bend toward the catalytic site, which is observable for all structures, making catalytic cysteine unavailable for substrate binding, as can be seen through the surface representations. Thus, the findings presented so far show great potential for the comprehension of human cancer theranostics through MALT1-targeted allosteric inhibition.

**Figure 12:**
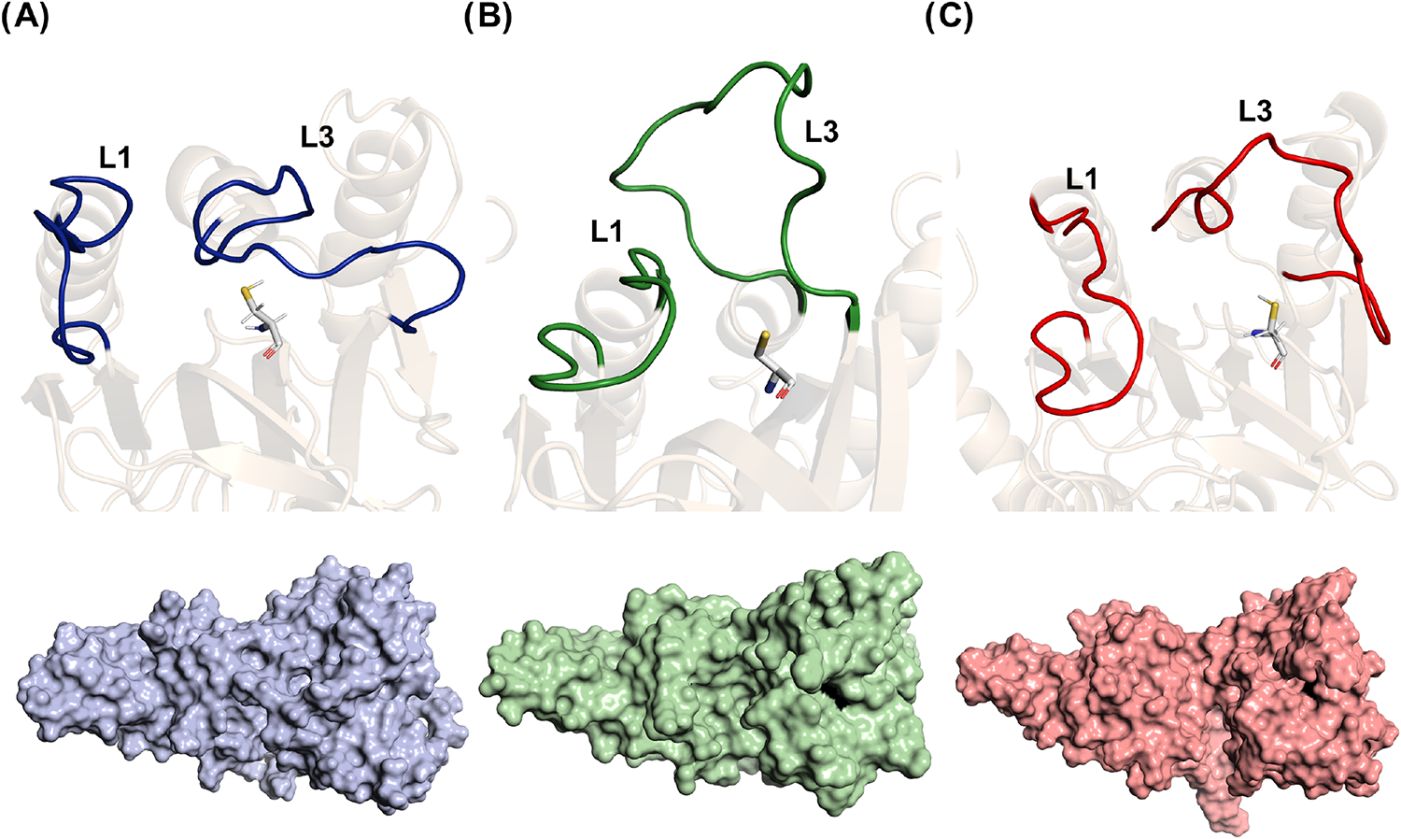
Comparison between the cartoon and surface representations of (A) meta 6 obtained through compound **1** simulations, (B) human MALT1 crystal PDB: 6F7I allosterically inhibited with MLT-747, and (C) meta 4 obtained through compound **2** simulations. In all cartoon representations, catalytic cysteine is shown in sticks, and in the surface representations, it is shown as a surface colored in black. In (A), the loops completely cover the catalytic cysteine, making it not observable.

### Covalent Docking for Inhibitory Ratio Evaluation

Despite observing loops 1 and 3, conformations are of great importance to obtain insights regarding CYS472 availability and MALT1 inhibition; covalent docking calculations with substrate mimic are crucial to confirm the hypothesis from structural observations and the expectations about compounds **1** and **2**. Therefore, for all collected representative structures for both compound-related systems, covalent docking calculations were performed to evaluate CYS472 availability and obtain the inhibition ratio for both compounds. As the substrate mimic, the covalent inhibitor Z-VRPR-fmk was used, and the docking energies are reported in Table 6 for compounds **1** and **2**.

**Table 6:** Calculated Docking Energies for Covalent Docking of Substrate Mimic Z-VRPR-fmk in Catalytic Cysteine (CYS472) of Metastable States’ Representative Structures Obtained from Compounds **1** and **2** Generated Distribution

	docking energy (kcal/mol)	
meta ID	compound **1**	compound **2**
0	–0.2	36.2
1	0.5	–0.4
2	91.7	–2.6
3	–2.5	81.2
4	–2.3	37.3
5	75.3	–
6	66.4	–
7	–2.6	–
8	1.1	–
9	–0.9	–
10	0.8	–
11	1.0	–

From Table 6, it is possible to observe that the mentioned metas of the compound **1** system, presenting a more pronounced loop 1 and 3 bending toward CYS472 (2, 5, and 6), presented very high docking energies, ranging from 66.4 to 91.7 kcal/mol. On the other hand, for the other metastable states (0, 1, 3, 4, 7, 8, 9, 10, and 11), much lower energies were obtained, ranging from −2.5 to 1.1 kcal/mol. Using as a benchmark the previously developed work by Santos et al.,^23^ in which MALT1 allosteric inhibited conformations through the usage of the commercially available MLT-748 allosteric inhibitor were investigated, the article showed that inhibited conformations presented a covalent docking energy higher than 19.6 kcal/mol, and the noninhibited conformations values lower than 19.6 kcal/mol. Therefore, using this value as the threshold for the decision making of inhibited and noninhibited conformations, and by observing Figure 13, it was possible to conclude that only metas 2, 5, and 6 were inhibited, as expected from conformational analysis. Therefore, compound **1**, when positioned in the allosteric pocket of MALT1, produces 29% of the inhibited structures, implying a high steric hindrance for substrate binding to CYS472 in these metas.

**Figure 13:**
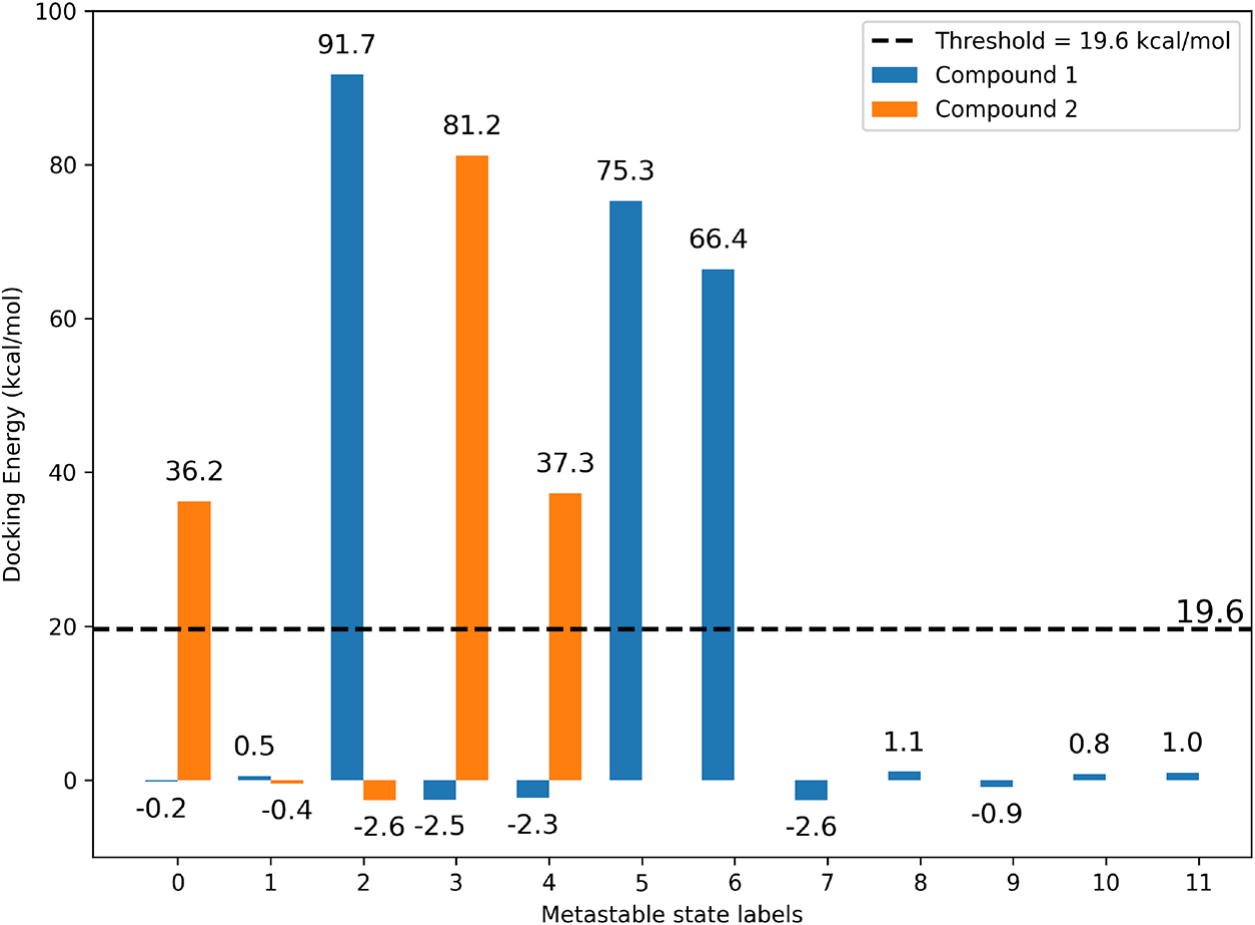
Docking energies for metastable states of the compounds **1** and **2** related models. The black dashed line corresponds to the threshold value of 19.6 kcal/mol, which was set by using the previously published work by Santos et al.^23^ as benchmark. Values higher than the threshold are considered inhibited states, and lower than the threshold, noninhibited states.

It is important to mention that compound **1** was initially designed to act as an ESIPT fluorescent probe only.^50^ However, from an inhibitory evaluation, a 29% inhibition ratio was observed, in which, despite presenting a lower inhibition ratio compared to MLT-748 (96.2%),^23^ it is substantially higher than when considering the clean MALT1, which presented no CYS472 unavailability.^23^ This occurred not only because some molecules were positioned in the MALT1 allosteric pocket but also probably due to the proposed molecule’s phenothiazine portion, which presents a very similar structure to promazine, an already reported potential MALT1 allosteric inhibitor.^29,75^

Now, for compound **2**’s inhibitory assessment, from Table 6, just as observed previously, the observed conformations from compound **2** distribution that presented a more aggressive loop 1 and 3 bending toward CYS472 (metastructures 0, 3, and 4) showed higher docking energies, ranging from 36.2 to 81.2 kcal/mol. On the other hand, conformations with less bent loops 1 and 3 (metas 1 and 2) presented much lower docking energies, ranging from −2.6 to −0.4 kcal/mol. Therefore, by using the same threshold and by observing Figure 13, it was possible to conclude that compound **2** generated a great majority of MALT1 catalytic loops in an inhibited state, implying a very dense catalytic site, in which the inhibition ratio totals 71.0% of the labeled data.

The inhibitory evaluation of compound **2** showed that by modifying the phenothiazine part of compound **1** using the thiorizadine structure, a much higher inhibition ratio was obtained, changing from 29.0% for compound **1** to 71.0% for compound **2**. Therefore, the inhibition ratio more than doubled when using compound **2** as a theranostic agent for MALT1 signaling and inhibition. Hence, the presented strategy proved to be very promising when seeking double-functional agents. The preservation of its probe capacity together with the substantial improvement in its inhibitory ratio shows that compound **2** is capable of both signaling and allosterically inhibiting the MALT1 target. These are crucial features when aiming for cancer diagnosis and therapy targeting this important cancer-related biomarker.

## Conclusion

The present work shows that by combining biased MD simulations with sophisticated neural network architectures, such as the CVAE architecture, a powerful framework is obtained to investigate protein inhibition through disordered loop conformational diversity and evaluate the ESIPT on/off distribution. From this, it was shown that the use of phenothiazine-benzothiazole derivatives presents a promising action both for cancer therapy and diagnosis through allosterically targeting the MALT1 protein. With this chemical structure combination, a unique compound can present both an ESIPT on/off distribution and spectroscopy capable of differentiating chemical environments when signaling, as well as a promising MALT1 inhibition capability.

It was observed that the benzothiazole portion is crucial to provide spectroscopic properties for compounds, and a protein environment enol emission in the blue region appears. This important feature is capable of differentiating chemical environments through ESIPT fluorescence, acting as a fingerprint for diagnostic purposes. These results highlight the critical role of enol emission in the evaluation of ESIPT-based probes for biological target signaling. This finding represents an important contribution to the field, as previous studies have largely focused on keto emissions as the primary parameter for distinguishing chemical environments. Regarding this scenario, compound **2** showed a higher fraction of ESIPT off conformations, at 40%, making it better for signaling purposes when compared with compound **1**, which showed a blue emission population of 28%. In addition, the phenothiazine portion showed a promising inhibitory capability even when using the compound **1** structure, and a simple modification in this portion was able to substantially increase the MALT1 inhibitory ratio from 29 to 71%, an increase of more than double. Therefore, from the performed modification generating an optimized structure for cancer theranostics through MALT1 targeting, compound **2** is shown to be a promising theranostic agent, being capable of both a high inhibition ratio and a substantial difference in ESIPT off spectroscopy between environments.

Thus, from this work, it was possible not only to provide a promising theranostic agent against cancer but also to provide a powerful theoretical framework for the proposal of ESIPT-based compounds for such a task, a technique that can extend to several biological systems using protein targets. However, it is important to emphasize that the use of a single representative conformation for the static covalent docking calculations, adopted to avoid prohibitively expensive computational costs, does not capture the structural heterogeneity arising from metastable states, and the authors acknowledge this limitation. Therefore, future studies should consider multiple representative conformations to provide a more comprehensive characterization of the intrastate variability. Nevertheless, the systematic application of this approach across different systems enables not only the determination of individual binding metrics but also the identification of meaningful trends associated with structural modifications of MALT1 allosteric inhibitors, thereby supporting the conclusions presented in this work.

## Supplementary Material



## Data Availability

GROMACS is available at https://www.gromacs.org/. PLUMED is available at https://www.plumed.org/. Binary contact maps data set, dihedrals data set, CVAE trained weights, COLVAR and DELTAFS files, simulation inputs, and data analysis codes are available at https://zenodo.org/records/18734376?token=eyJhbGciOiJIUzUxMiJ9.eyJpZCI6ImE5OTcxMjVkLWU2MDQtNGQ1NC1hYzA2LWEwNmI4ZmVhNmM5MCIsImRhdGEiOnt9LCJyYW5kb20iOiI2OTFiNjExM2QxNGIxMGY0ZWJjMWZjNDg4OWIxZjljOSJ9.Yqa46Rsu8FwgglvwE-Z3Po2349JscA2mGgUcVSAGWAG-tkmKVfk99QKzOOBq4BvWWLzgsy-CsSeB_TuLMCzkNg.

## Notes

ESIPT-related statistics and spectrum analog, CVAE training profile, calculated additional metrics, and external validation map reconstruction images (PDF)
