# Supplementary material for: From Dynamics to Diagnosis and Therapy: A Multiscale Computational Framework for MALT1-Targeted Cancer Theranostics
Source: J Chem Inf Model. 2026 Jul 30;66(15):9365–84. doi: 10.1021/acs.jcim.6c01276 (PMC13471727; doi:10.1021/acs.jcim.6c01276)
Supplement: Supplementary file 1 [file ci6c01276_si_001.pdf]

SUPPORTING INFORMATION: From Dynamics to Diagnosis and Therapy: A Multiscale Computational Framework for MALT1-Targeted Cancer Theranostics

Rodrigo M. Santos† and Teodorico C. Ramalho*,†,‡

†*Laboratory of Molecular Modelling, Department of Chemistry, Federal University of Lavras, Lavras 37200-000, Minas Gerais, Brazil*

‡*Centre for Basic and Applied Research, Faculty of Informatics and Management, University of Hradec Králové, Hradec Králové 500 03, Czech Republic*

E-mail: rodrigomancini4@gmail.com,teo@ufla.br

SI. 1 - Biased MD Convergence

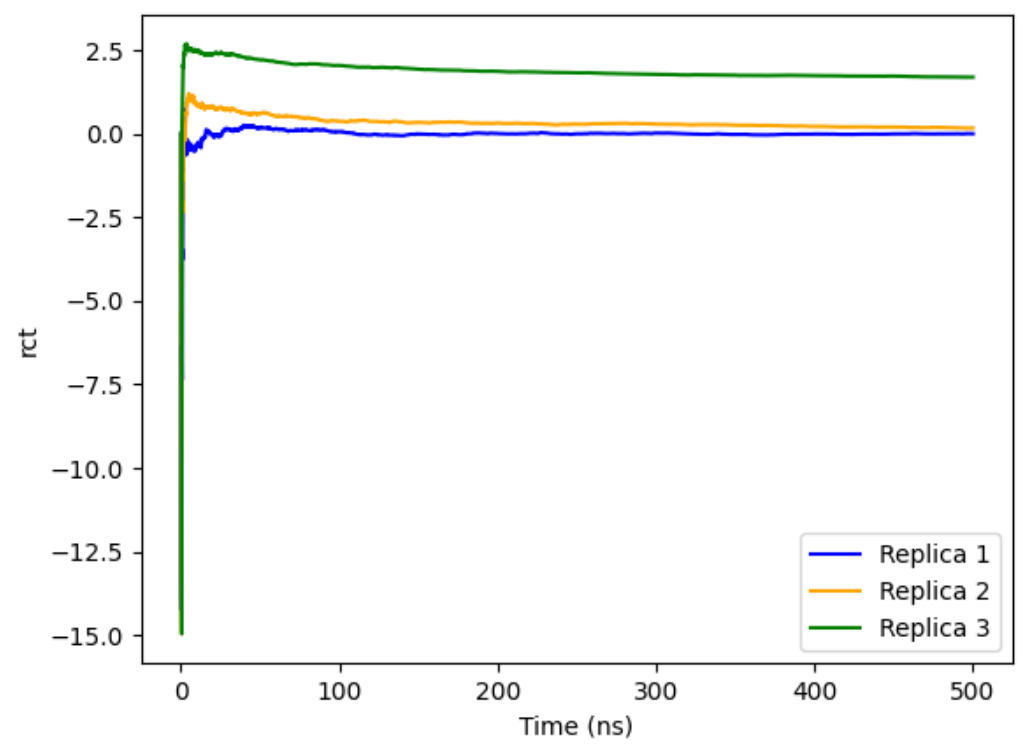

(a)

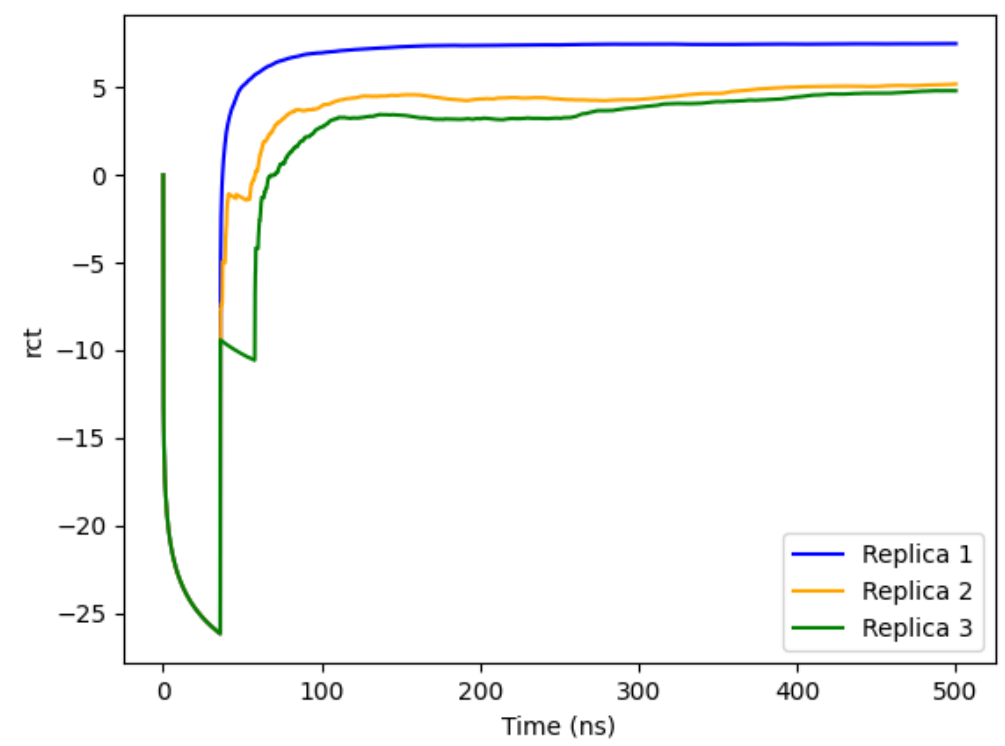

(b)

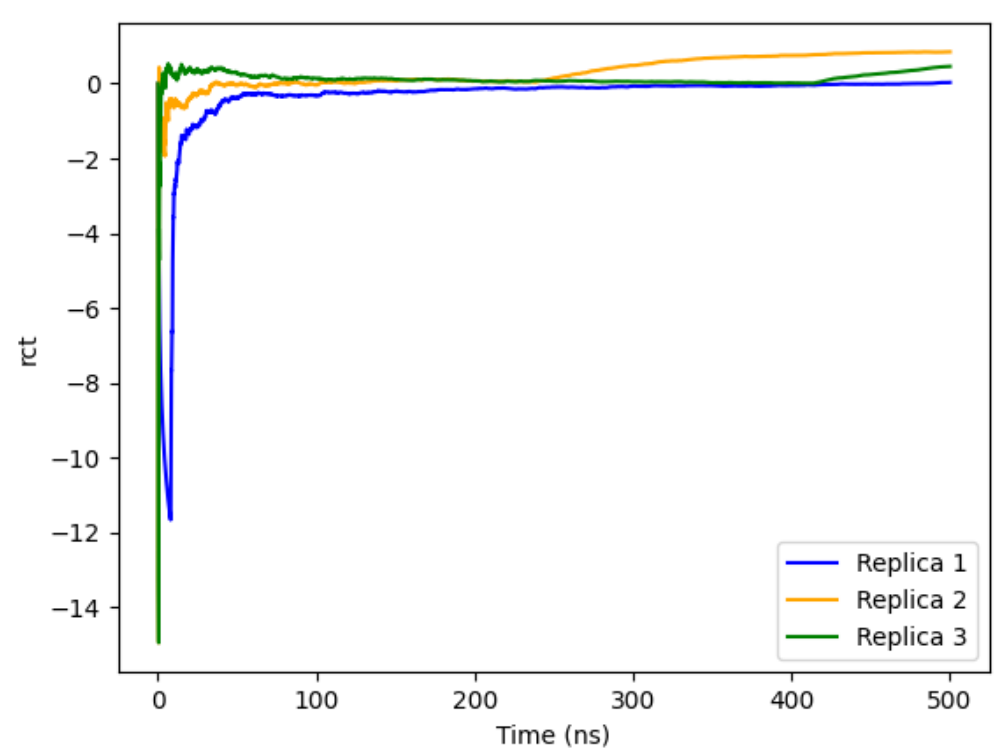

(c)

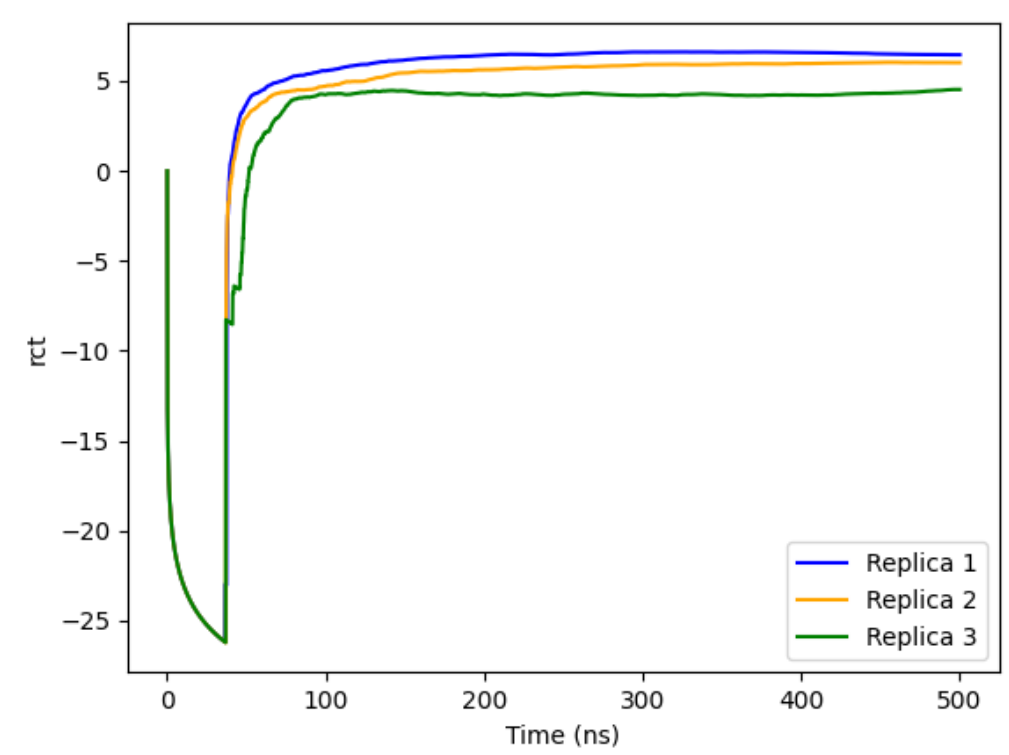

(d)

Figure S1: Biased MD convergence profiles of all replicas through reaction coordinate (rct) vs time in systems a) compound **1** in water only, b) compound **1** in protein, c) compound **2** in water only, and d) compound **2** in protein.

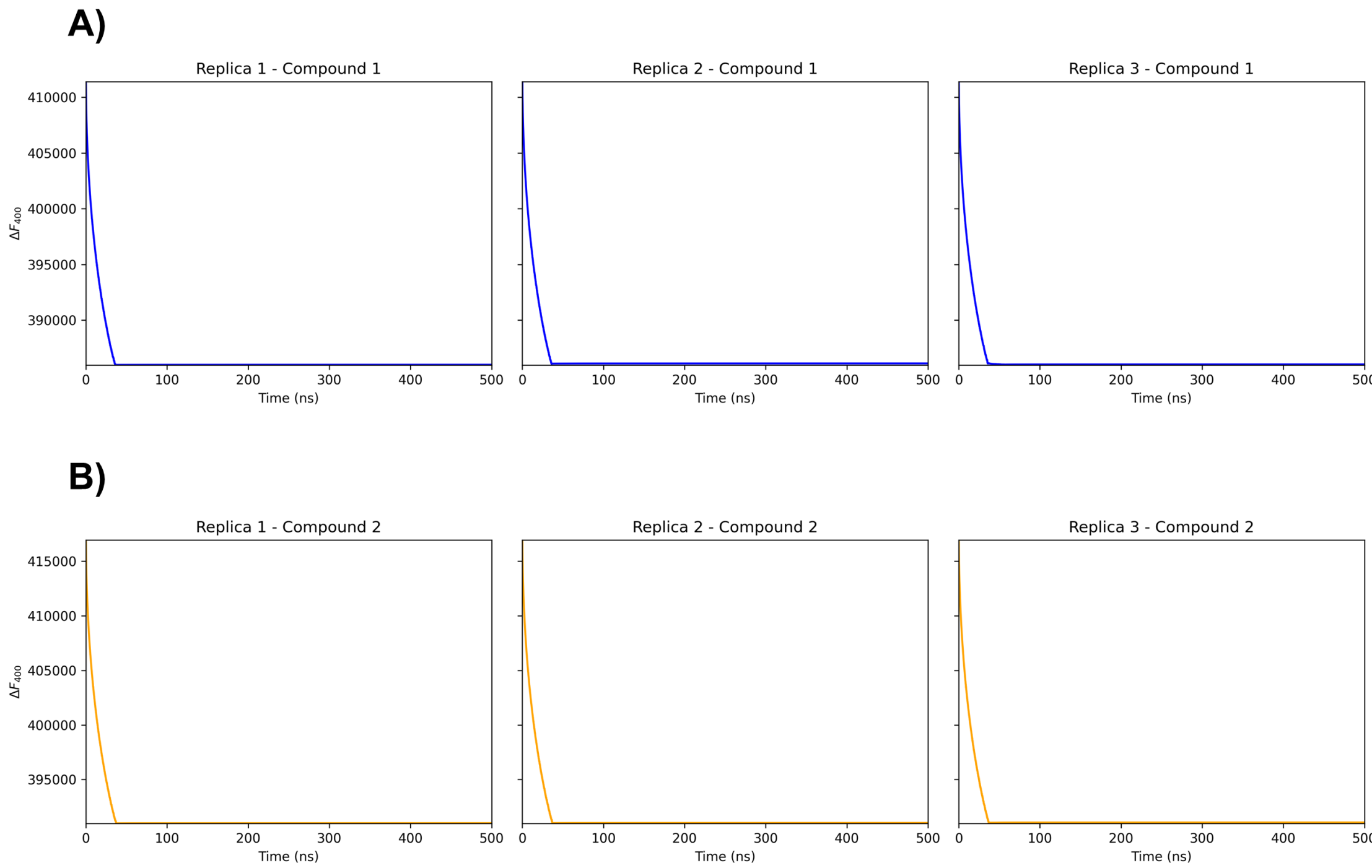

Figure S2: Free-energy difference associated with reweighting the ensemble from the reference temperature (300 K) to 400 K for (A) compound **1** system, and (B) compound **2** system.

Table S1: Block analysis (50 ns intervals) of the multithermal free-energy estimate ΔF_{400} for the three independent OPES replicas of compound **1** system. Mean values and standard deviations (SD) of ΔF_{400} were computed within each time block. After approximately 100 ns, ΔF_{400} exhibited a stationary behavior characterized by negligible change of the mean value and dramatical decrease of the standard deviations across consecutive blocks.

Block (ns)	Replica 1		Replica 2		Replica 3	
	Mean	SD	Mean	SD	Mean	SD
0–50	391673.780	6224.631	391770.817	6230.563	391763.394	6234.813
50–100	385988.779	1.035	386102.461	2.205	386019.892	3.917
100–150	385990.889	0.387	386105.801	0.456	386023.486	0.445
150–200	385991.943	0.227	386106.683	0.101	386024.291	0.155
200–250	385992.610	0.173	386107.284	0.170	386024.901	0.175
250–300	385993.157	0.143	386107.684	0.128	386025.771	0.324
300–350	385993.582	0.104	386108.329	0.226	386026.651	0.189
350–400	385993.942	0.104	386109.052	0.206	386027.244	0.168
400–450	385994.267	0.091	386109.545	0.093	386027.875	0.143
450–500	385994.555	0.079	386109.885	0.106	386028.298	0.130

Table S2: Block analysis (50 ns intervals) of the multithermal free-energy estimate ΔF_{400} for the three independent OPES replicas of compound **2** system. Mean values and standard deviations (SD) of ΔF_{400} were computed within each time block. After approximately 100 ns, ΔF_{400} exhibited a stationary behavior characterized by negligible change of the mean value and dramatical decrease of the standard deviations across consecutive blocks.

Block (ns)	Replica 1		Replica 2		Replica 3	
	Mean	SD	Mean	SD	Mean	SD
0–50	397022.241	6398.229	396995.412	6366.054	397016.536	6372.025
50–100	391002.652	0.965	391024.288	0.929	391065.235	1.825
100–150	391004.896	0.468	391026.463	0.512	391067.908	0.363
150–200	391006.149	0.274	391027.788	0.248	391068.693	0.159
200–250	391006.929	0.164	391028.562	0.205	391069.251	0.167
250–300	391007.526	0.169	391029.195	0.166	391069.779	0.100
300–350	391007.995	0.110	391029.704	0.110	391070.177	0.135
350–400	391008.343	0.094	391030.098	0.100	391070.510	0.102
400–450	391008.608	0.066	391030.459	0.104	391070.868	0.126
450–500	391008.809	0.063	391030.763	0.070	391071.301	0.149

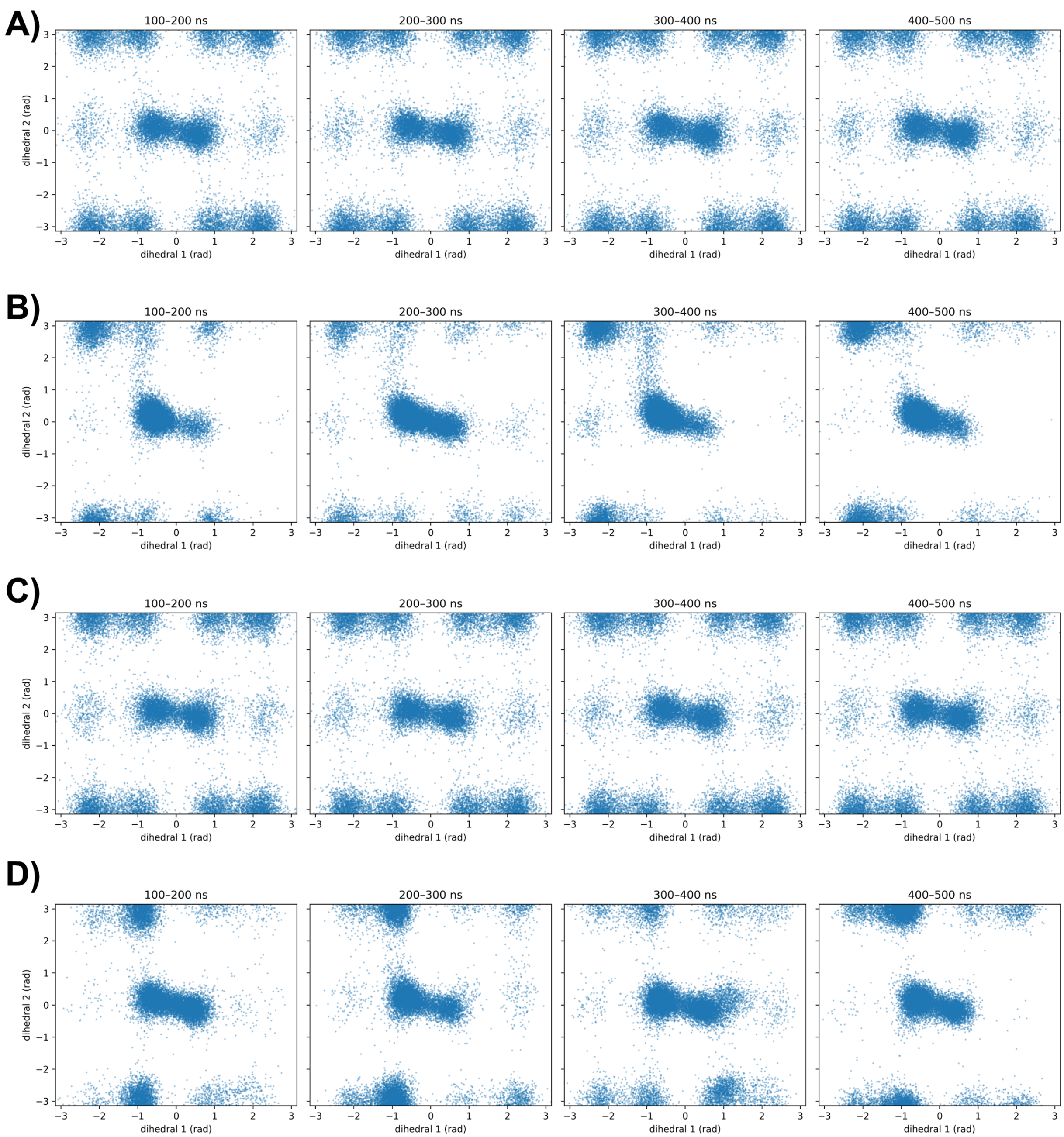

Figure S3: Dihedral's distribution in blocks of 100 ns, discarding the block ranging from 0 to 100 ns to observe only after the bias equilibration period. (A) for compound **1** in water only, (B) for compound **1** in protein, (C) for compound **2** in water only, (D) for compound **2** in protein. For all systems, a consistent dihedral distribution through the simulation evolution was observed, implying the achievement of a stationary regime.

SI. 2 - QM Protein Chemical Environment

Table S3: Collected amino acid residues in the representative meta conformation obtained through esipt on/off distribution assessment. The two closest residues of -OH and -N= group of the benzothiazole portion was considered for the QM calculations.

Meta #	Collected Amino Acid Residues	
	Compound **1**	Compound **2**
1	GLU405/ARG584	MET725/ASN401
2	ALA721/GLN587	ARG727/ASP724
3	ILE720/VAL583	GLU405/ASN401
4	GLU405/ILE720	ARG727/ASN401
5	ILE720/ARG584	MET725/ASN401
6	GLU398/GLN684	HIS726/ASN401
7	LYS687/GLN587	MET725/ARG727
8	ASN401/ARG727	MET725/ASN401
9	ILE720/ARG584	ILE720/ASN401
10	ALA402/ASN401	LEU723/ARG727
11	LYS687/LEU723	ASP724/ASN401
12	LYS687/GLN587	MET725/ASN401

SI. 3 - Compound 1 Evaluation Related Information

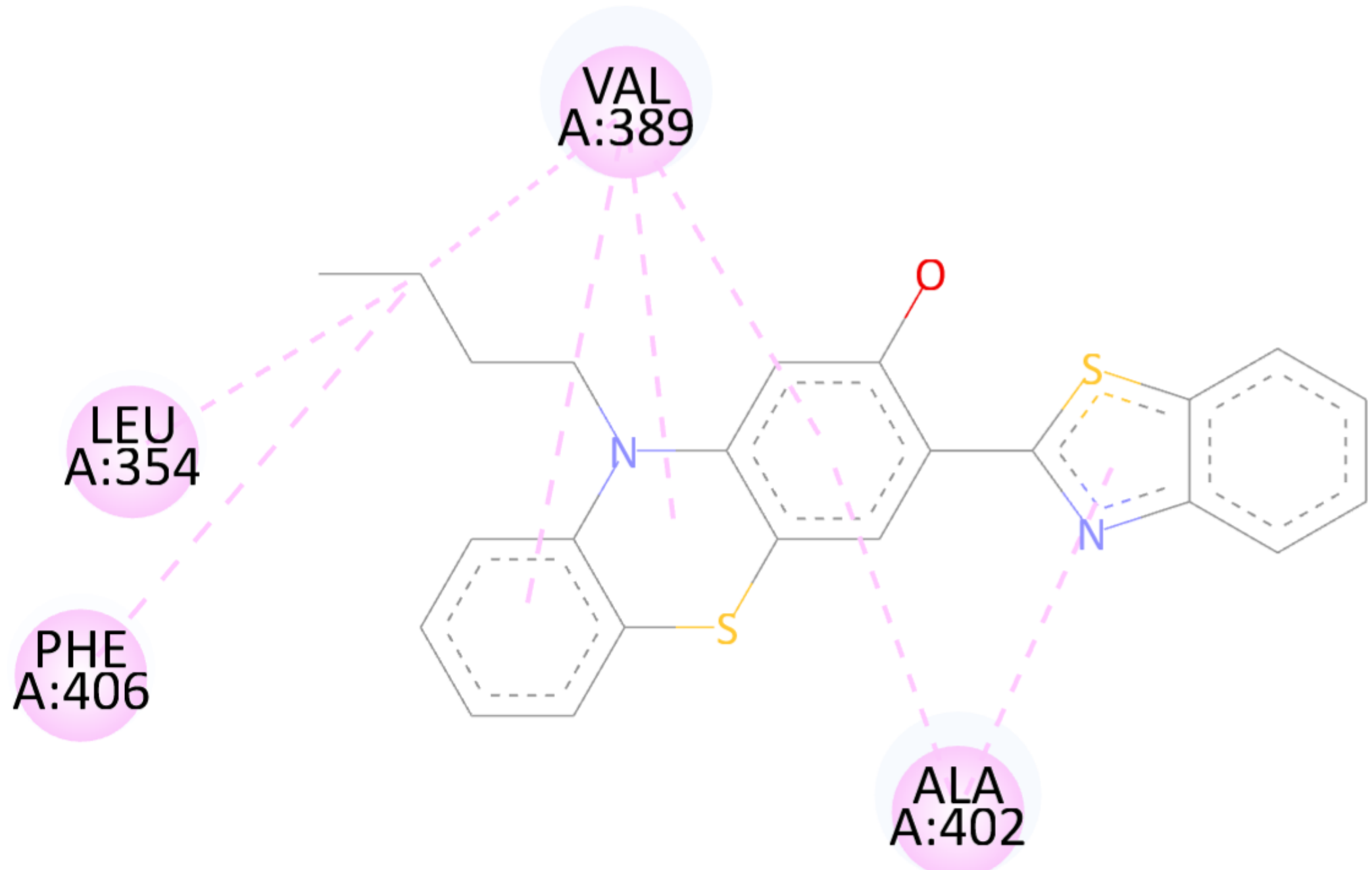

Figure S4: Reported initial structure interactions between compound **1** and the MALT1 allosteric pocket, generated by BIOVIA Discovery Studio 2024.[1]

Table S4: Calculated hbond distance metrics between the -OH group and -N= group of the benzothiazole portion for compound 1.

Meta #	Water Only Env.		Protein Env.	
	Mean hbond (Å)	std (Å)	Mean hbond (Å)	std (Å)
1	4.953286	0.211549	4.943182	0.196492
2	4.168177	0.193799	4.205057	0.204732
3	4.041311	0.206943	4.024487	0.174620
4	4.866534	0.242305	4.889762	0.244465
5	3.681769	0.266819	3.768762	0.252517
6	2.164835	0.241626	2.128136	0.175798
7	2.159600	0.234547	2.054056	0.150867
8	3.662017	0.279565	3.651357	0.297755
9	4.867249	0.239999	4.866747	0.218351
10	4.043184	0.210669	3.997152	0.247598
11	4.175952	0.197428	4.092591	0.170599
12	4.961272	0.203325	4.944226	0.218190

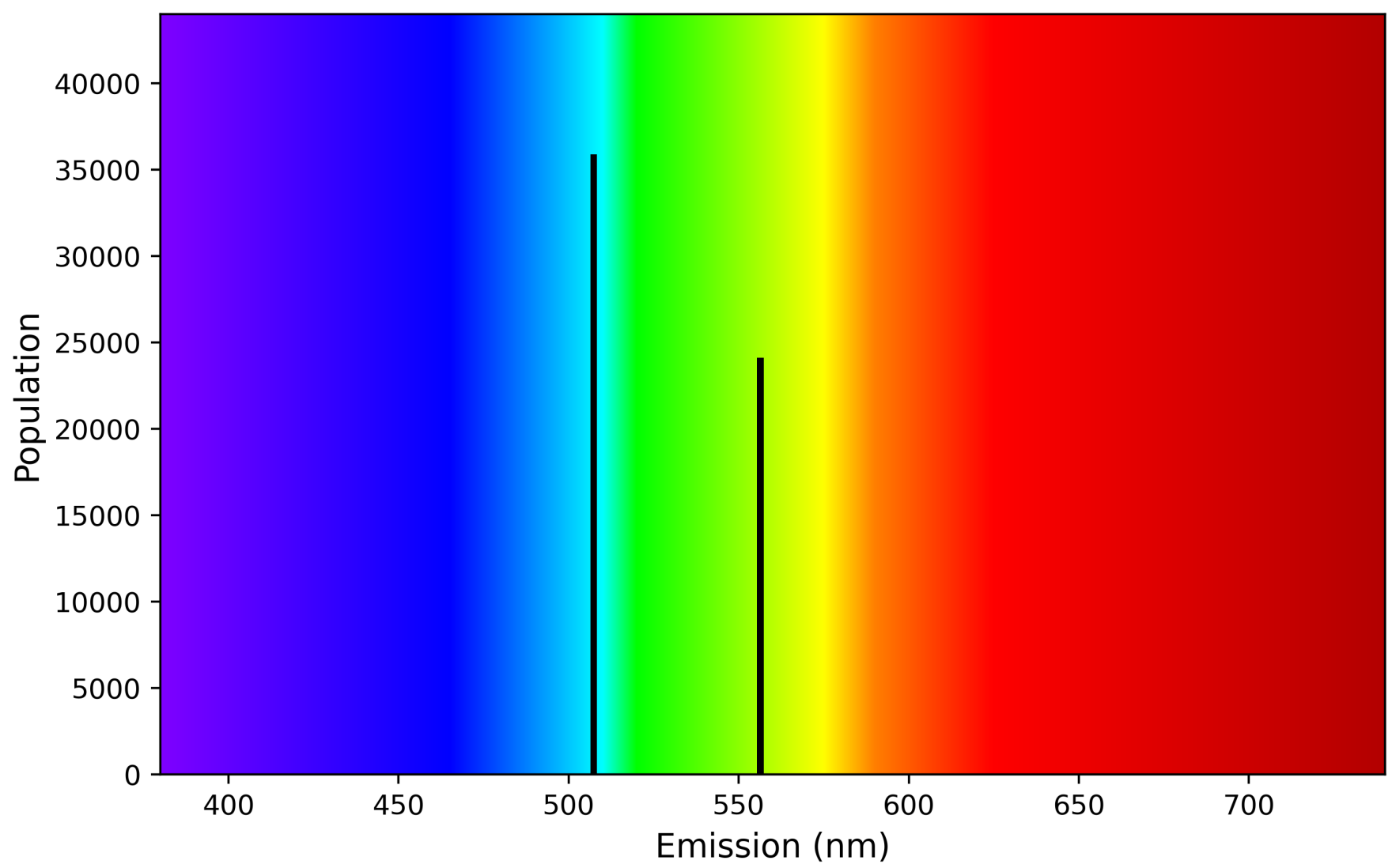

(a)

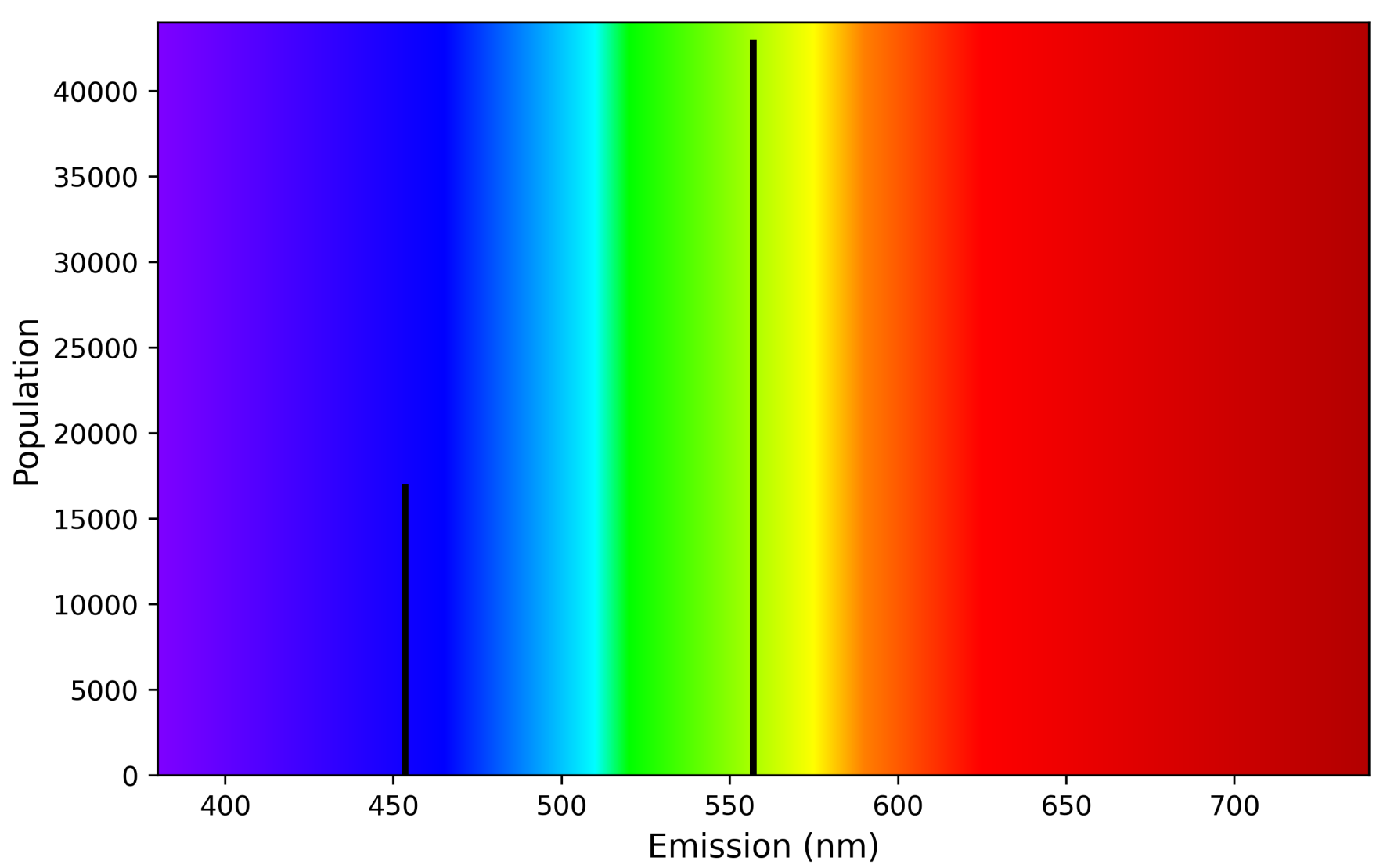

(b)

Figure S5: Emission spectrum analog of compound **1**, built using the population count of configurations with enol and keto emission and their average wavelength emission in a) the water-only environment and b) the protein environment.

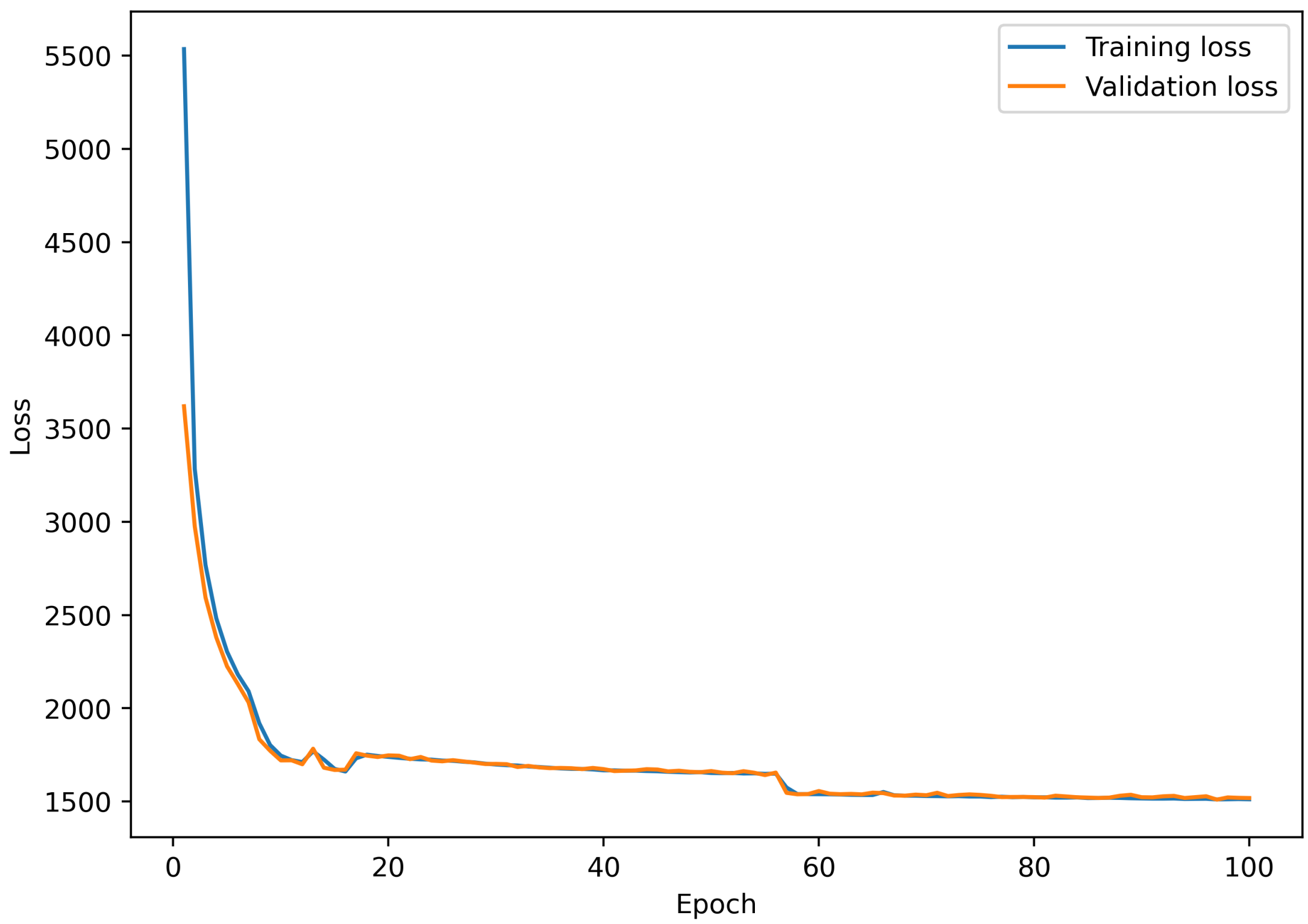

Figure S6: Obtained training profile curve for the CVAE used for compound 1 evaluation.

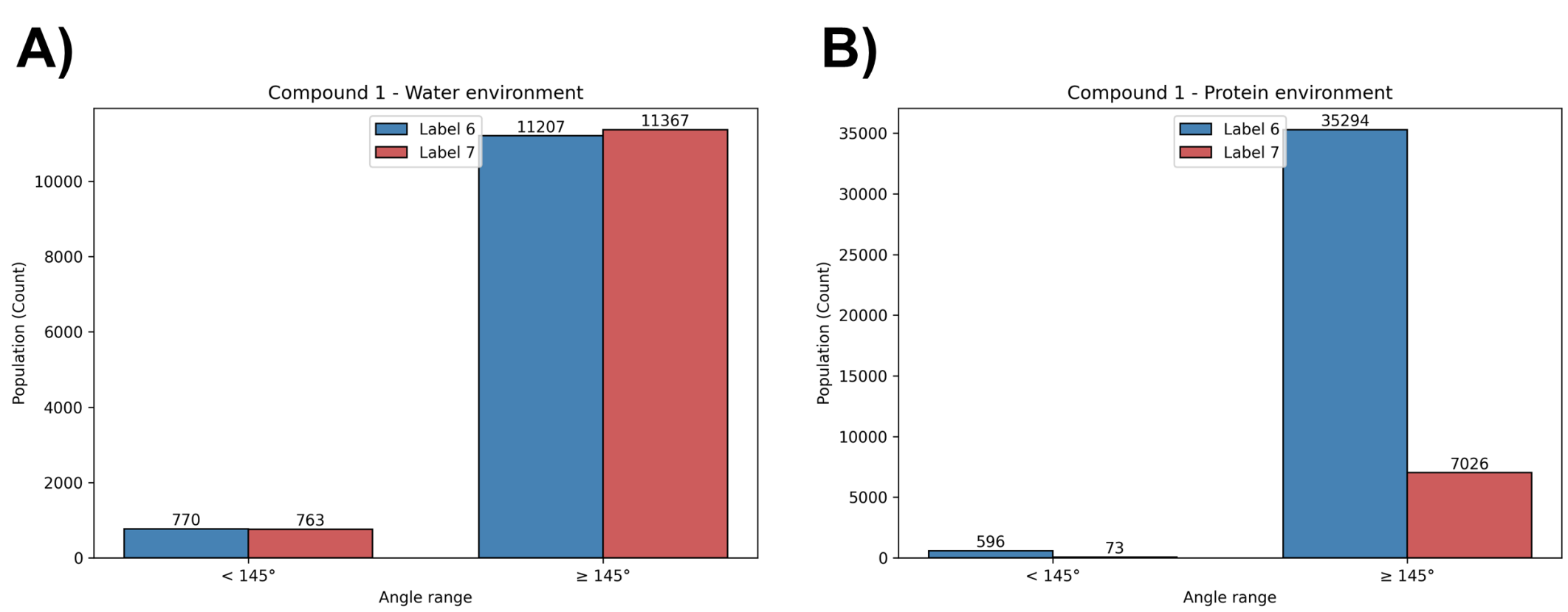

Figure S7: Obtained compound **1** angle distributions for (A) water environment and (B) protein environment. The angle used as a cutoff was 145°, once this value corresponded to the optimal angle donor-hydrogen-acceptor through b3lyp/6-311g QM calculation. Considering the angle distribution, an ESIPT ON/OFF of 38%/62% was obtained in a water environment, and 71%/29% was obtained for the protein environment; hence, a negligible deviation was observed when compared to only using the distance criteria.

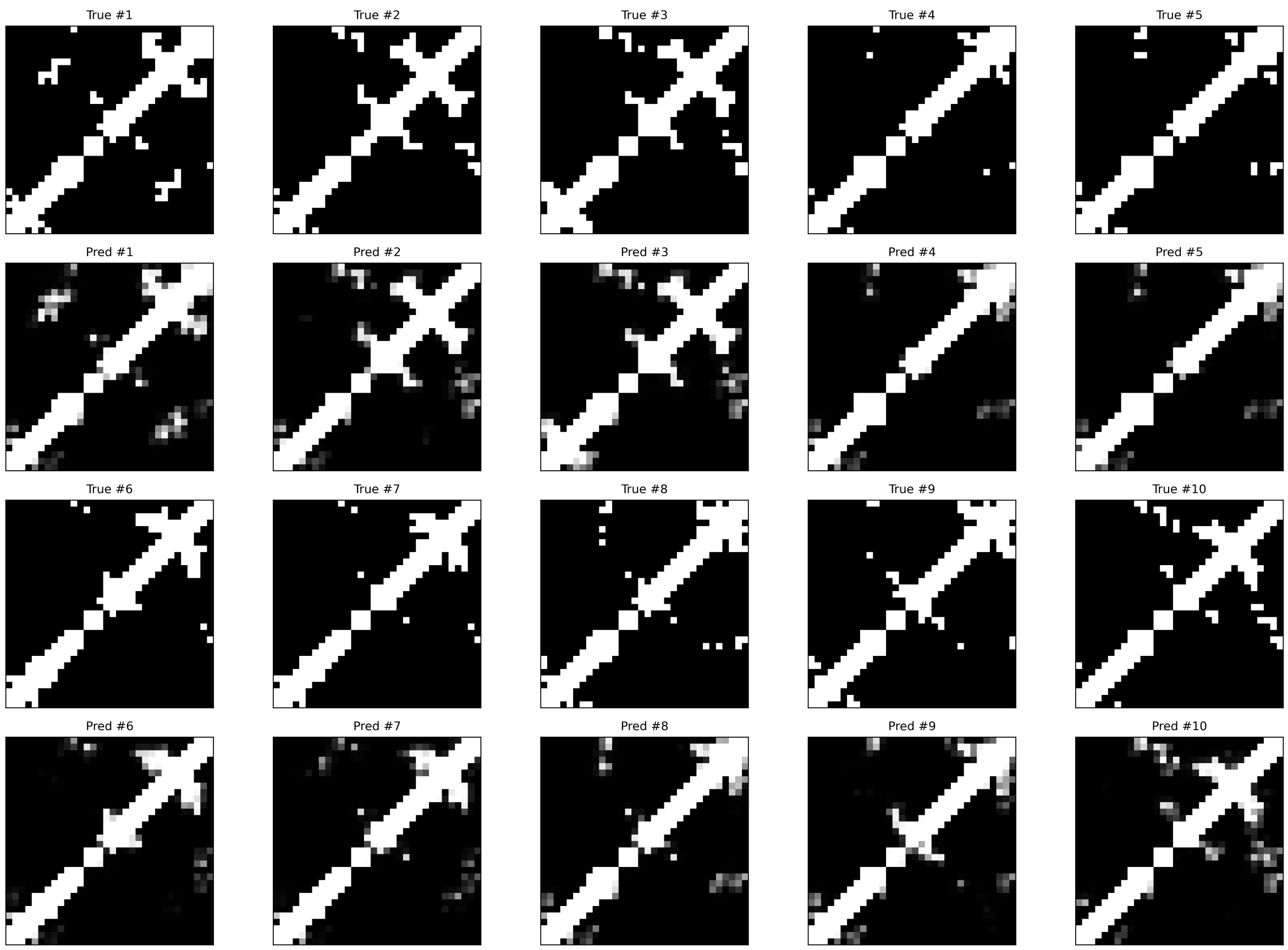

Figure S8: True and reconstructed maps used as external validation for compound 1 evaluation.

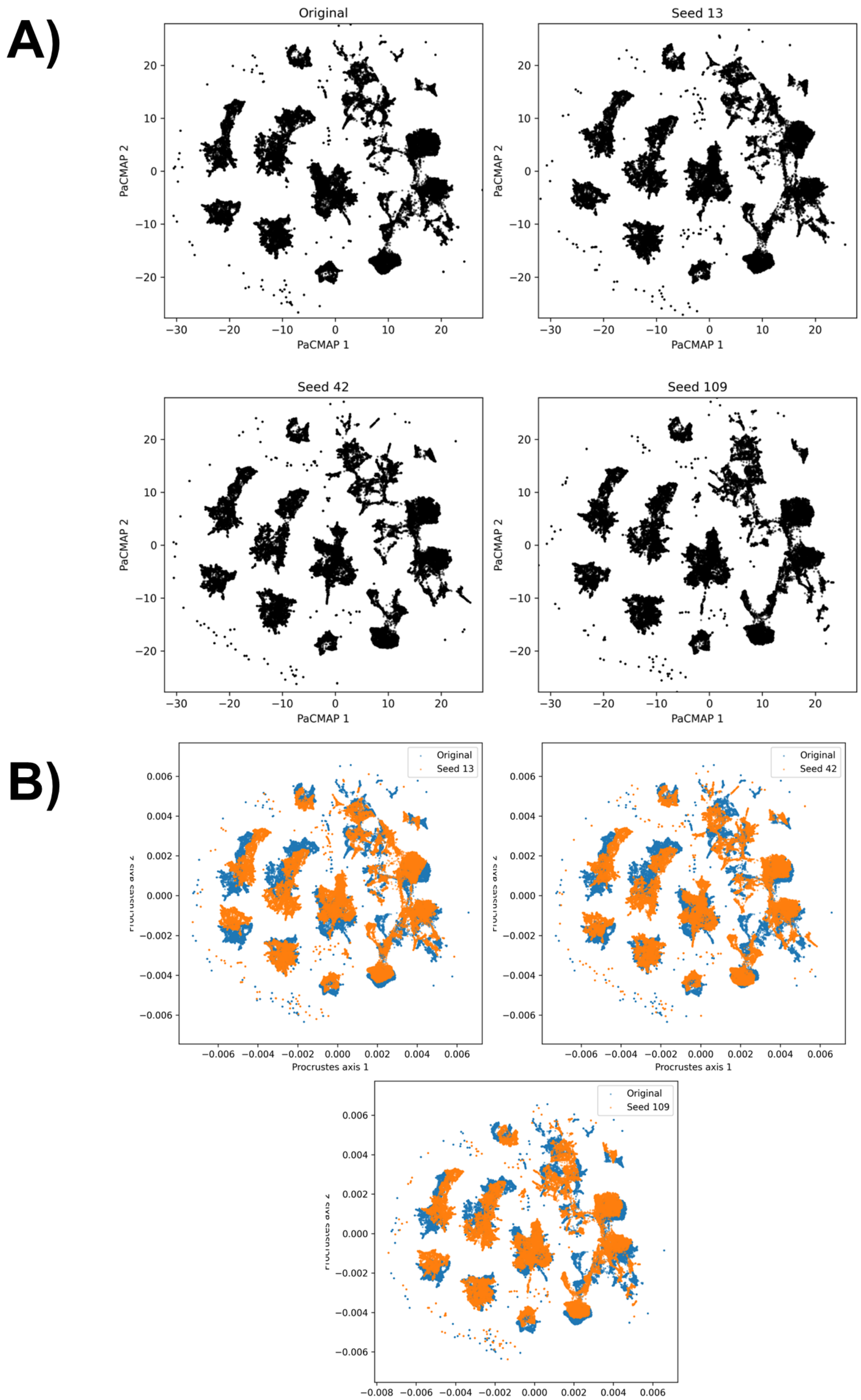

Figure S9: Procrustes analysis for the compound **1** model. (A) Shows raw PACMAP spaces of the original space and the ones built from seeds 13, 42, and 109. (B) Shows the aligned PACMAP spaces of the seeds using as reference the original PACMAP space.

SI. 4 - Compound 2 Evaluation Related Information

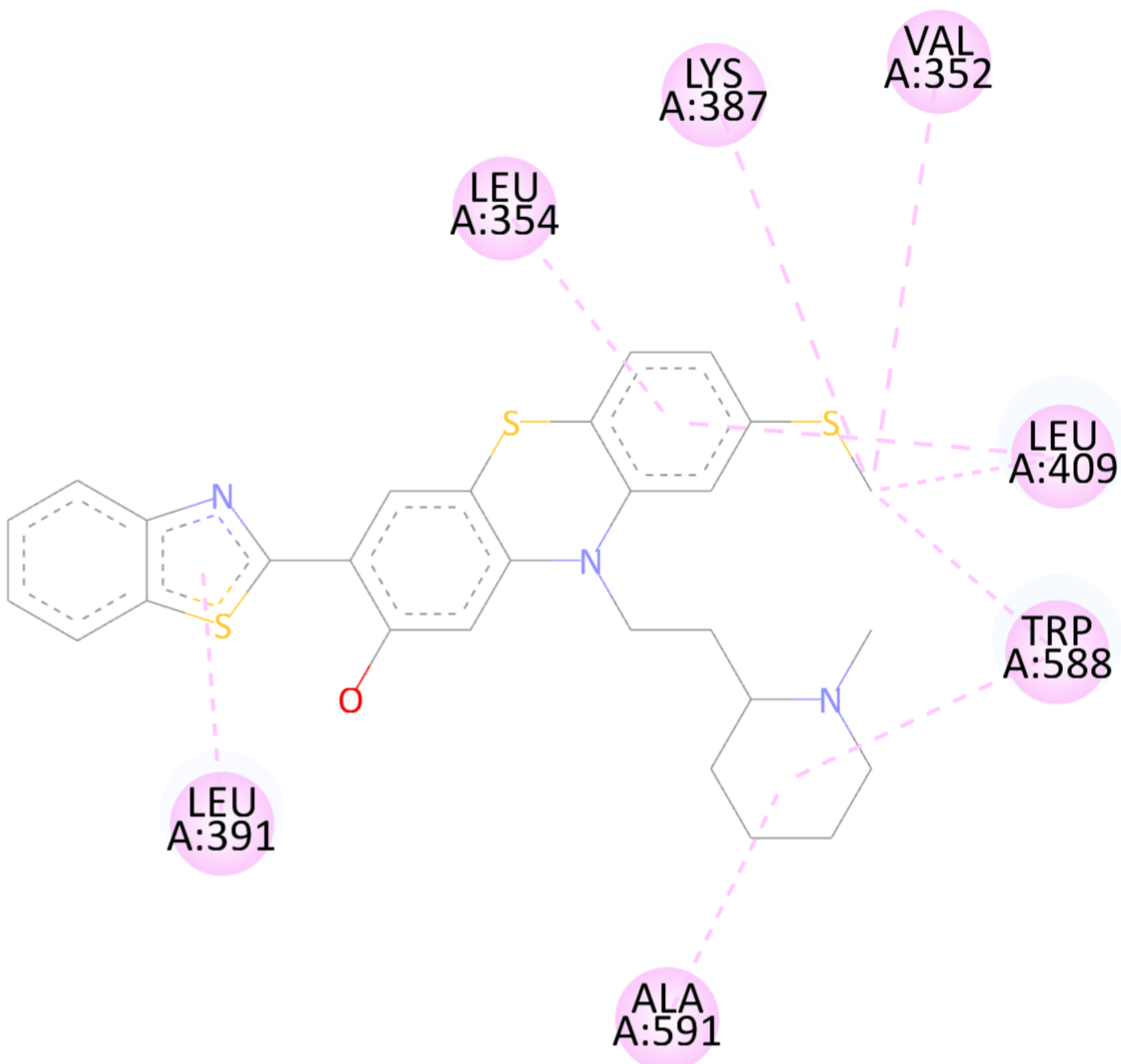

Figure S10: Reported initial structure interactions between compound **2** and the MALT1 allosteric pocket, generated by BIOVIA Discovery Studio 2024.[1]

Table S5: Calculated hbond distance metrics between the -OH group and -N= group of the benzothiazole portion for compound 2.

Meta #	Water Only Env.		Protein Env.	
	Mean hbond (Å)	std (Å)	Mean hbond (Å)	std (Å)
1	4.958881	0.207096	4.893478	0.223331
2	4.175856	0.194846	4.141681	0.168657
3	4.051310	0.209632	4.002181	0.188182
4	4.870313	0.245149	4.814494	0.270249
5	3.673638	0.274350	3.608450	0.310025
6	2.163133	0.236319	2.145445	0.203995
7	2.162828	0.237343	2.100759	0.230526
8	3.674703	0.273068	3.540254	0.323322
9	4.865106	0.244897	4.761237	0.256044
10	4.054636	0.214981	4.005472	0.187933
11	4.177577	0.195496	4.140221	0.197449
12	4.962626	0.211368	4.931751	0.213925

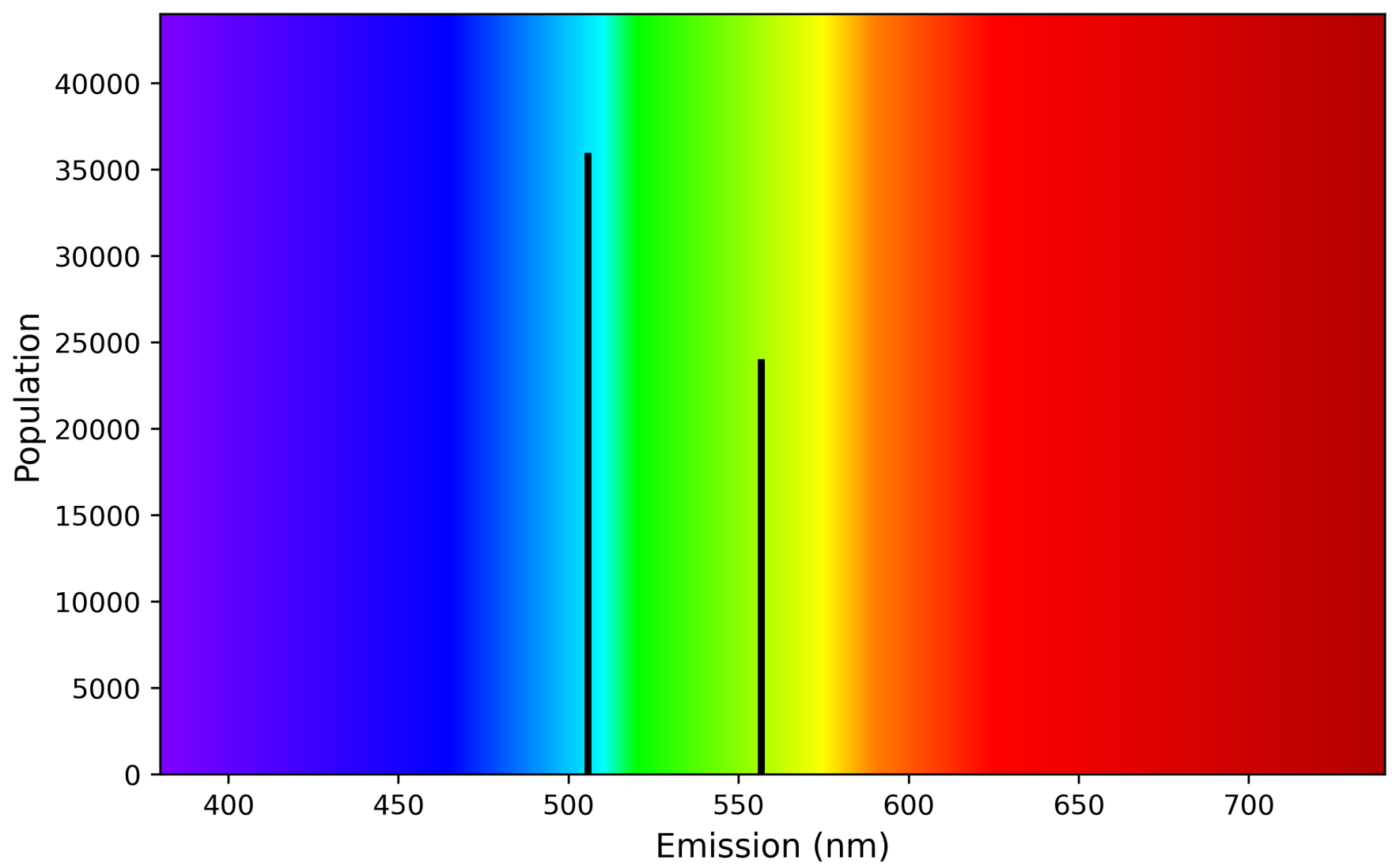

(a)

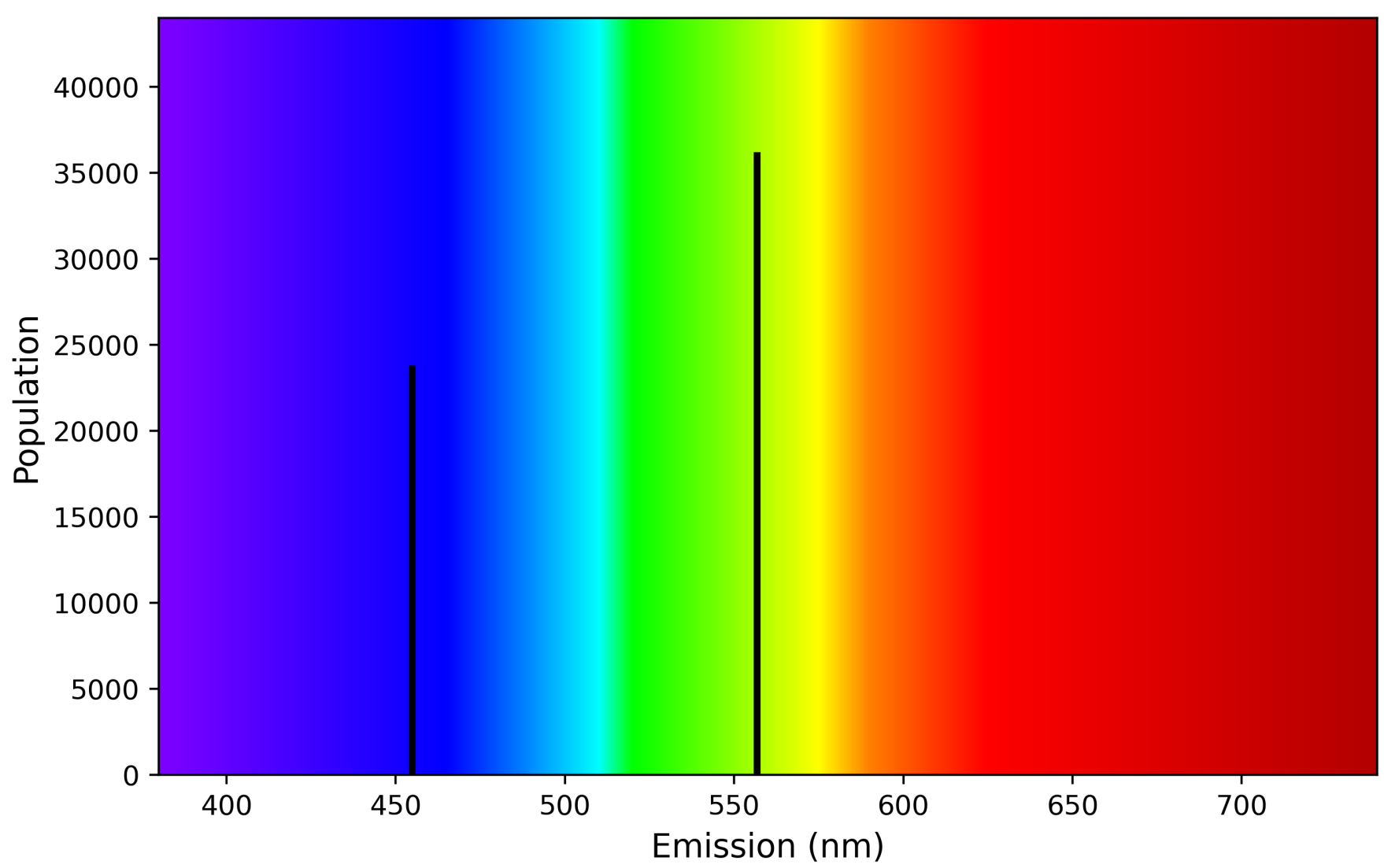

(b)

Figure S11: Emission spectrum analog of compound **2**, built using the population count of configurations with enol and keto emission and their average wavelength emission in a) the water-only environment and b) the protein environment.

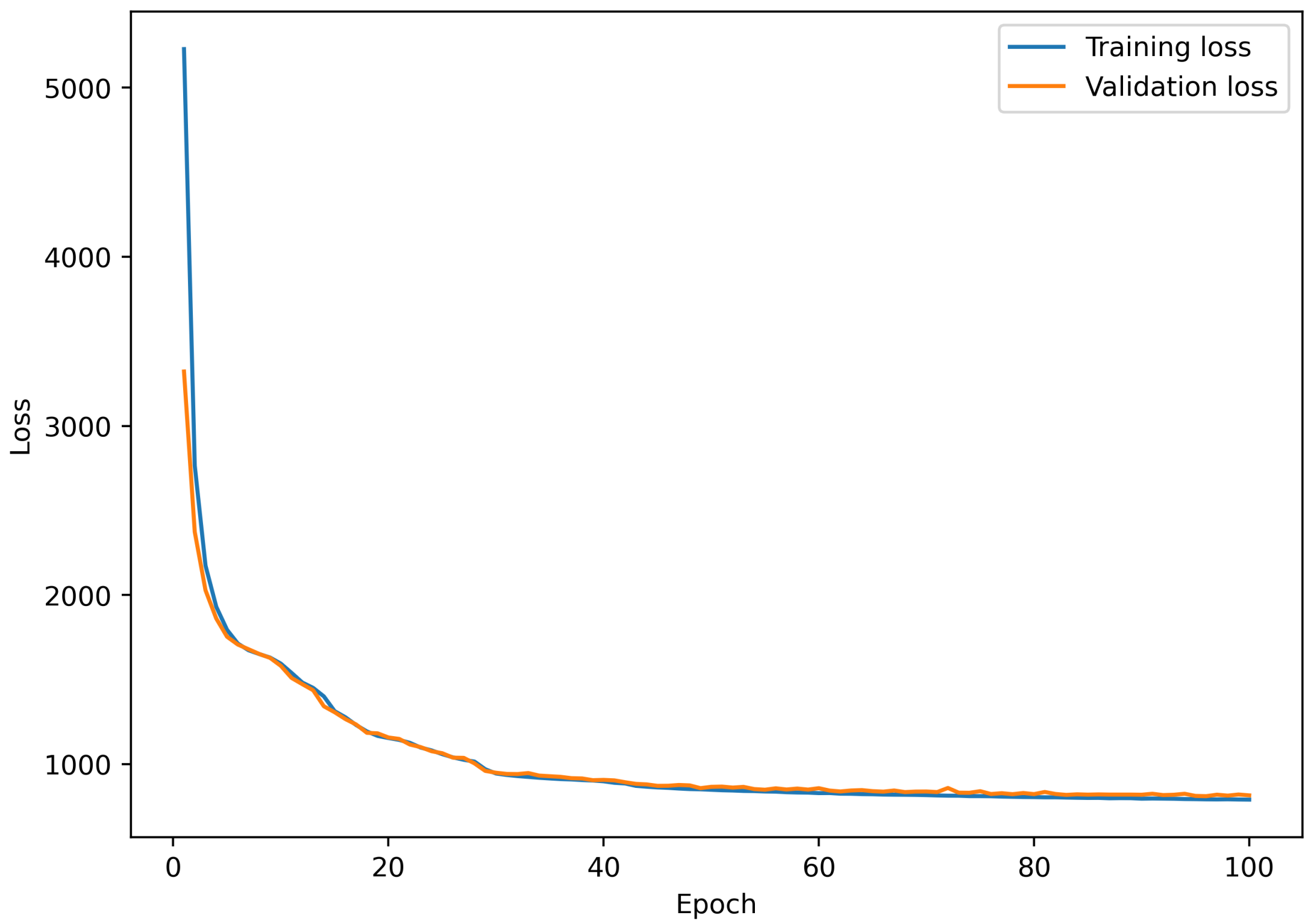

Figure S12: Obtained training profile curve for the CVAE used for compound 2 evaluation.

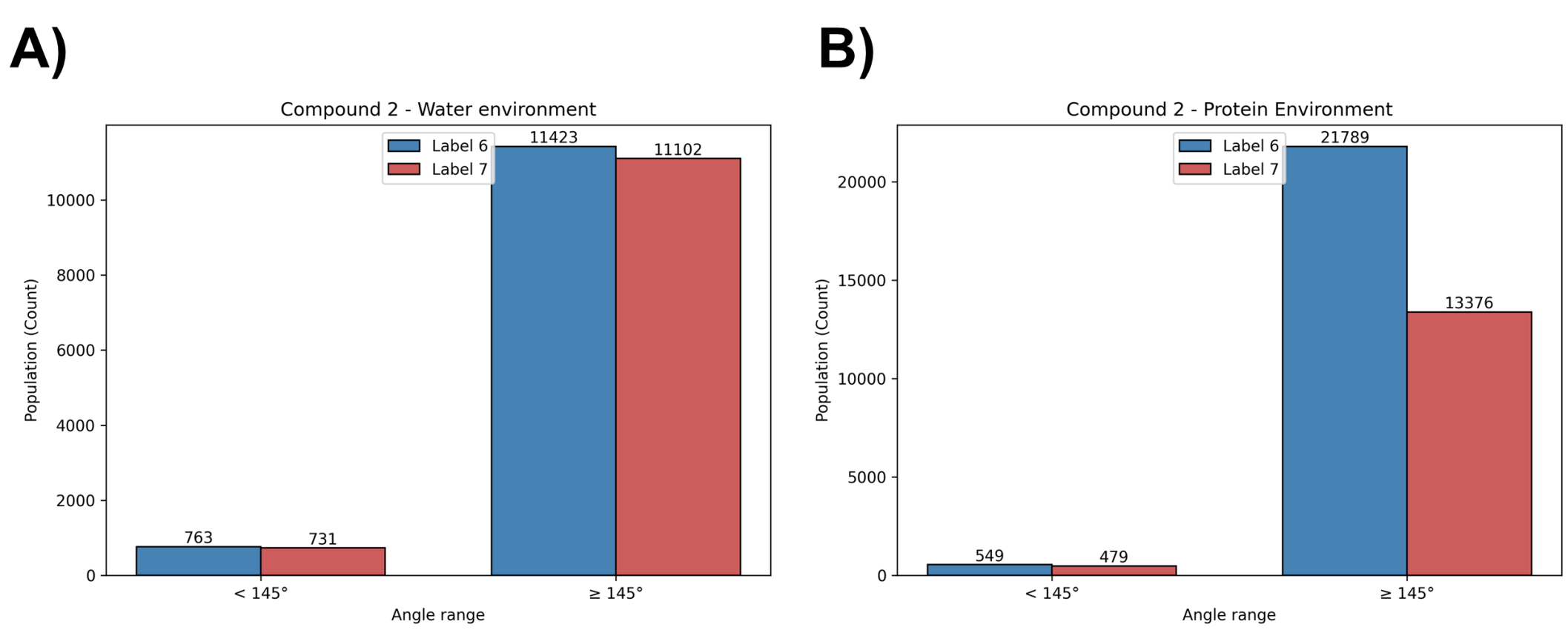

Figure S13: Obtained compound **2** angle distributions for (A) water environment and (B) protein environment. The angle used as a cutoff was 145°, once this value corresponded to the optimal angle donor-hydrogen-acceptor through b3lyp/6-311g QM calculation. Considering the angle distribution, an ESIPT ON/OFF of 38%/62% was obtained in a water environment, and 59%/41% was obtained for the protein environment; hence, a negligible deviation was observed when compared to only using the distance criteria.

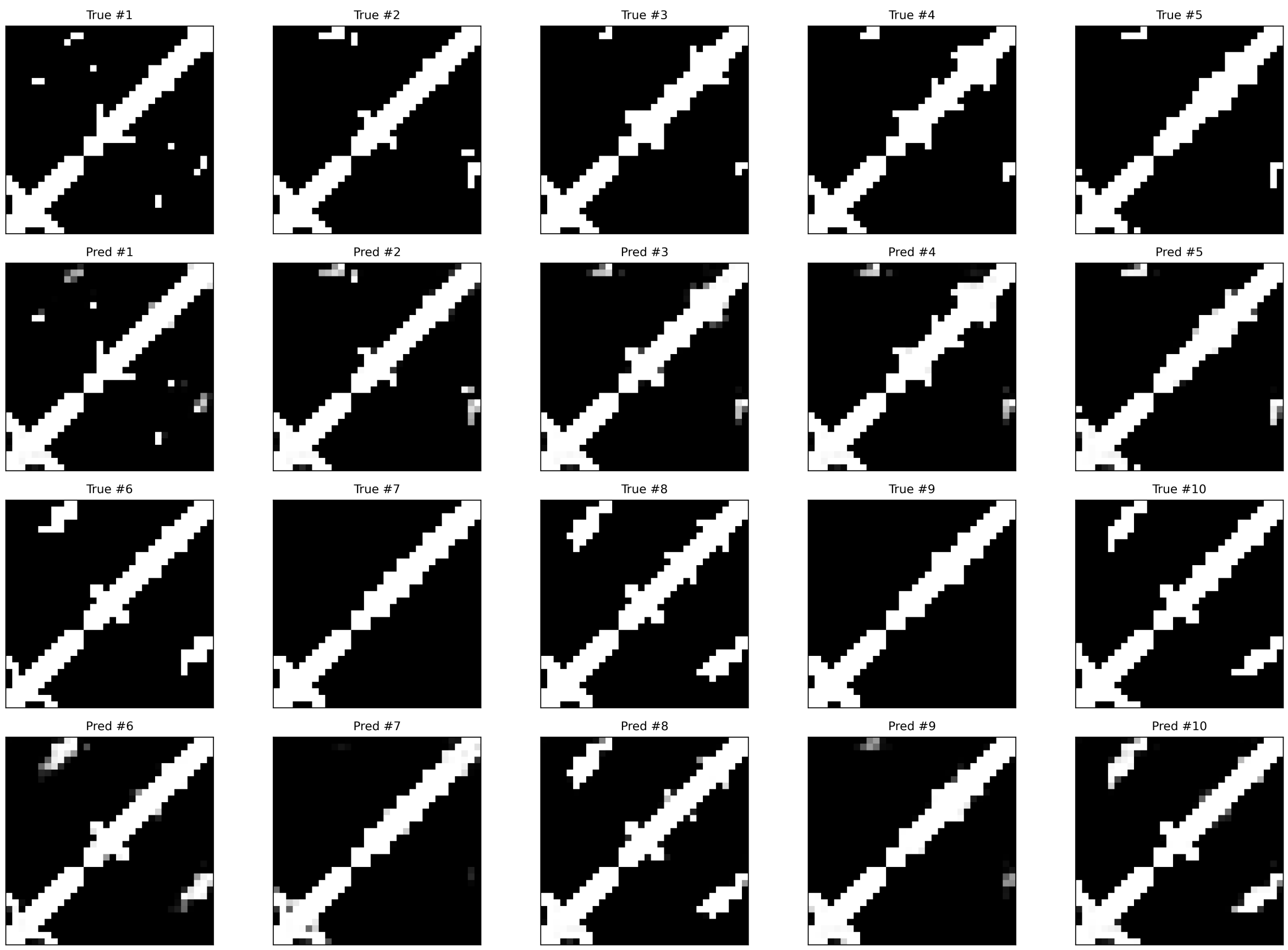

Figure S14: True and reconstructed maps used as external validation for compound 2 CVAE.

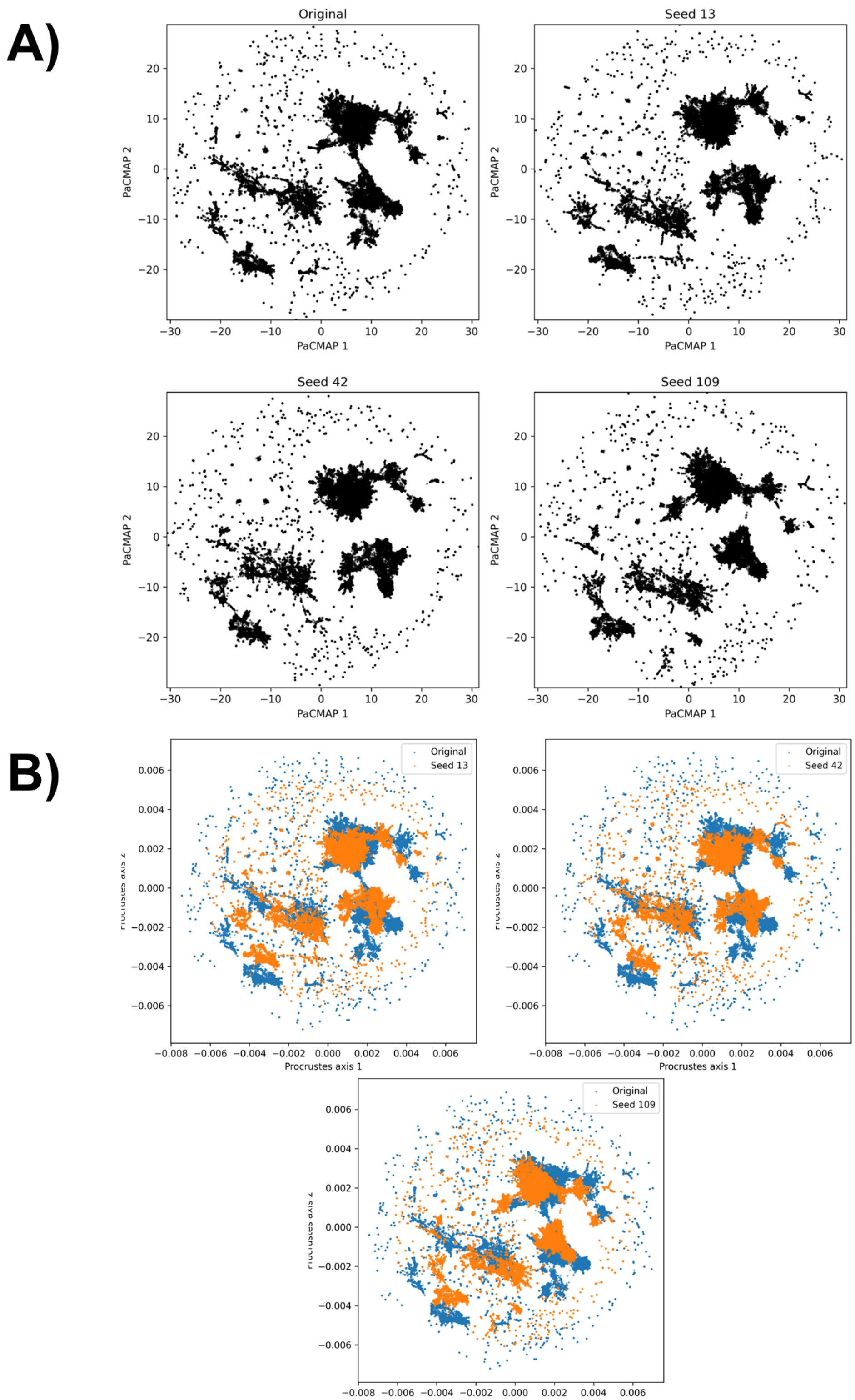

Figure S15: Procrustes analysis for the compound **2** model. (A) Shows raw PACMAP spaces of the original space and the ones built from seeds 13, 42, and 109. (B) Shows the aligned PACMAP spaces of the seeds using as reference the original PACMAP space.

SI. 5 - Full QM ESIPT spectroscopy

Table S6: Calculated emission wavelengths with TDDFT using full QM for both compounds. In column "Conformations", the compound is denoted as C1 for compound **1**, C2 for compound **2**, m5 for meta 5, and m6 for meta 6, as an identification of which compound conformation and meta were collected to perform the full QM calculation. The calculation was performed following the same procedure described in the methodology, but now replacing the usage of UFF for the chemical environment with the quantum level of theory. The presented results show very low deviation compared to the ones using ONIOM, and the same emission region was achieved (green for keto emission, cyan for water enol emission, and blue for protein enol emission).

Conformation	Water Env. Emission (nm)		Protein Env. Emission (nm)	
	Enol	Keto	Enol	Keto
C1-m5	516.77	-	-	-
C1-m6	-	-	-	559.12
C2-m5	-	-	469.18	-
C2-m6	-	558.56	-	-

References

(1) Dassault Systèmes BIOVIA BIOVIA Discovery Studio. 2024.
